# SCRABBLE: single-cell RNA-seq imputation constrained by bulk RNA-seq data

**DOI:** 10.1186/s13059-019-1681-8

**Published:** 2019-05-06

**Authors:** Tao Peng, Qin Zhu, Penghang Yin, Kai Tan

**Affiliations:** 1 Division of Oncology and Center for Childhood Cancer Research, Children’s Hospital of Philadelphia, Philadelphia, PA 19104 USA; 2 Graduate Group in Genomics and Computational Biology, University of Pennsylvania, Philadelphia, PA 19104 USA; 3 Department of Mathematics, University of California, Los Angeles, CA 90095 USA; 4 Department of Biomedical and Health Informatics, Children’s Hospital of Philadelphia, Philadelphia, PA 19104 USA; 5 Department of Pediatrics, Perelman School of Medicine, University of Pennsylvania, Philadelphia, PA 19104 USA; 6 Department of Cell and Developmental Biology, Perelman School of Medicine, University of Pennsylvania, Philadelphia, PA 19104 USA; 7 Department of Genetics, Perelman School of Medicine, University of Pennsylvania, Philadelphia, PA 19104 USA

**Keywords:** Single-cell RNA-seq, Imputation, Matrix regularization, Optimization

## Abstract

Single-cell RNA-seq data contain a large proportion of zeros for expressed genes. Such dropout events present a fundamental challenge for various types of data analyses. Here, we describe the SCRABBLE algorithm to address this problem. SCRABBLE leverages bulk data as a constraint and reduces unwanted bias towards expressed genes during imputation. Using both simulation and several types of experimental data, we demonstrate that SCRABBLE outperforms the existing methods in recovering dropout events, capturing true distribution of gene expression across cells, and preserving gene-gene relationship and cell-cell relationship in the data.

**Electronic supplementary material:**

The online version of this article (10.1186/s13059-019-1681-8) contains supplementary material, which is available to authorized users.

## Background

Single-cell RNA sequencing (scRNA-seq) has revolutionized cell biology, enabling studies of heterogeneity and transcriptome dynamics of complex tissues at single-cell resolution. However, a major limitation of scRNA-seq data is the low capturing and sequencing efficiency affecting each cell, resulting in a large proportion of expressed genes with zeros or low read counts, which is known as the “dropout” phenomenon. Such dropout events lead to bias in downstream analysis, such as clustering, classification, differential expression analysis, and pseudo-time analysis. To address this critical challenge, two types of approaches have been developed. One approach adopts analysis strategies that take dropout into consideration. For instance, ZINB-WaVE generates weights for genes and cells using a zero-inflated negative binomial model which in turn is used to detect differential expression [1]. Lun et al. used a pool-and-deconvolute approach to deal with dropout events for accurate normalization of scRNA-seq data [2]. The second approach is direct imputation of scRNA-seq data. Among these methods, MAGIC imputes dropout events by data diffusion based on a Markov transition matrix that defines a kernel distance measure among cells [3]. scImpute [4] first computes dropout probability using a two-component mixture model. It then uses a LASSO model to impute dropout values. Similarly, SAVER [5] also uses a linear regression to impute the missing data. But, it differs from the scImpute by using a Bayesian model to compute the probability of dropout events. DrImpute [6] first conducts consensus clustering of cells followed by imputation by the average value of similar cells. VIPER uses a non-negative sparse regression model to progressively infer local neighborhood cells for imputation [7].

All imputation methods above recover dropout values using scRNA-seq only. Here, we describe the SCRABBLE algorithm for imputing scRNA-seq data by using bulk RNA-seq as a constraint. SCRABBLE only requires consistent cell population between single-cell and bulk data. The bulk data represent the unfractionated composite mixture of all cell types without sorting them into individual types. For many scRNA-seq data, there are usually existing bulk data on the same cell/tissue. And it is becoming increasingly common to collect matched bulk data when a new scRNA-seq experiment is performed. Bulk RNA-seq data allows SCRABBLE to achieve a more accurate estimate of the gene expression distributions across cells than using single-cell data alone. SCRABBLE is based on the framework of matrix regularization that does not impose an assumption of specific statistical distributions for gene expression levels and dropout probabilities. It also does not force the imputation of genes that are not affected by dropout events.

## Results

**Fig. 1:**
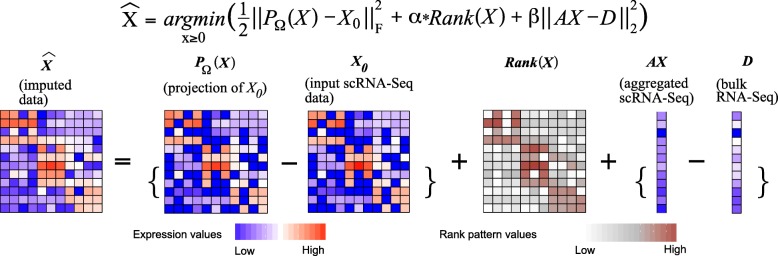
Schematic overview of the SCRABBLE algorithm. The objective function is shown on the top. It has three terms. The first term represents the difference between the raw scRNA-seq data matrix and its projection of the optimizing matrix. The projection of the optimizing matrix has the same profile of zeros as that of the raw scRNA-seq data. The second term is the rank of the optimizing matrix. The third term represents the difference between the bulk RNA-seq data and the aggregated scRNA-seq data across cells. Here, the bulk data represent the composite mix of all cell types without sorting them into individual types

SCRABBLE is based on the mathematical framework of matrix regularization [8]. It imputes dropout data by optimizing an objective function that consists of three terms (Fig. 1). The first term ensures that imputed values for genes with non-zero expression remain as close to their original values as possible, thus minimizing unwanted bias towards expressed genes. The second term ensures the rank of the imputed data matrix to be as small as possible. The rationale is that we only expect a limited number of distinct cell types in a given tissue sample. The third term operates on the bulk RNA-seq data. It ensures consistency between the average gene expression of the aggregated imputed data and the average gene expression of the bulk RNA-seq data. We developed a convex optimization algorithm to minimize the objective function (see the “Methods” section). The existence of an optimal solution is guaranteed mathematically [8].

**Fig. 2:**
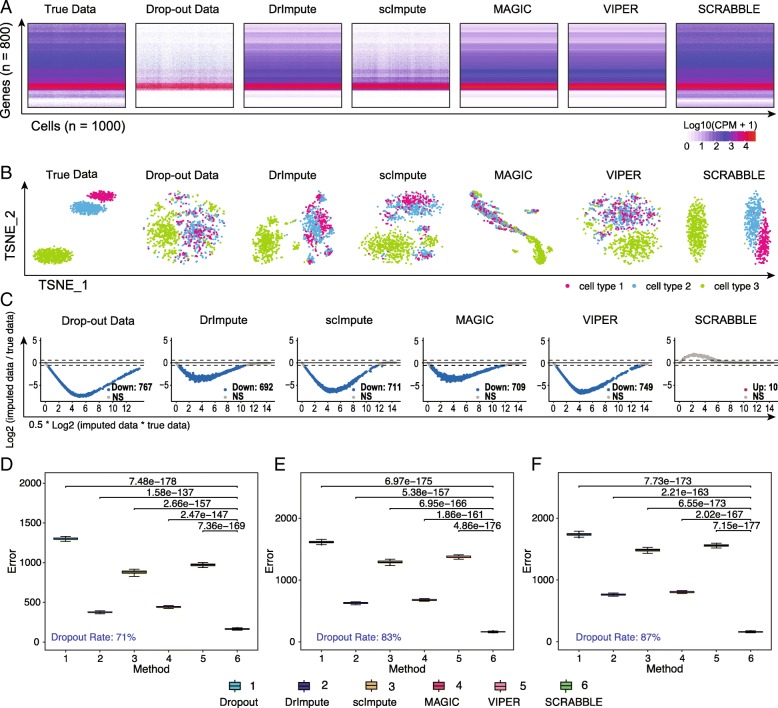
Performance evaluation using synthetic data. **a** A representative imputation result using simulated data containing 1000 cells and 800 genes. The data was simulated using the Splatter method [9]. The dropout rate is 83%. **b** t-SNE plots of the representative imputation results. **c** MA plots of the representative imputation results. **d**–**f** Imputation errors for data with different percentages of zeros in the data (71%, 83%, and 87%). The imputation error is defined as the *L*_2_ norm of the difference between the imputed data matrix and the true data matrix. Each boxplot represents the result from 100 simulated datasets. *P* values are based on Student’s *t* test

**Fig. 3:**
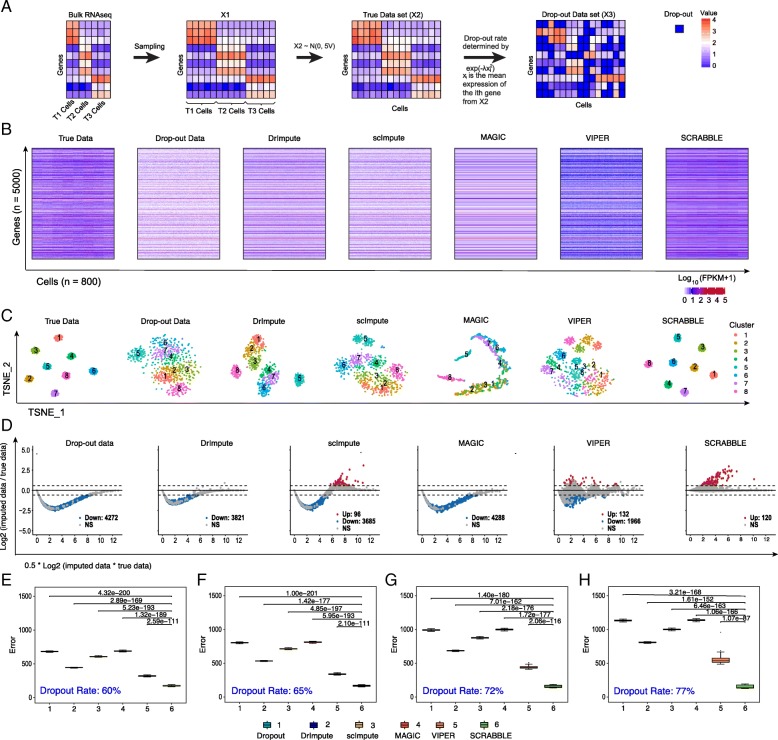
Performance evaluation using down-sampled bulk RNA-seq data. **a** Schematic overview of the simulation strategy. Starting from the bulk RNA-seq data matrix consisting of three types of cells, T1 cells, T2 cells, and T3 cells, the data matrix *X*_1_ is obtained by resampling of raw data from the different type cells separately. Then, each element (*x*_*ij*_) in the data matrix is perturbed by the normal distribution *N*(0, 5*V*) (*V* is the vector of standard deviation of genes across replicates in the bulk RNA-seq data), and the true data set *X*_2_ is generated. Finally, dropout events are introduced in *X*_2_ using an exponential function, resulting in the dropout data set *X*_3_. **b** A representative imputation result using simulated data. The dropout rate is 72%. **c** t-SNE plots of the representative imputation results. **d** MA plots of the representative imputation results. Imputation errors for data with 60% (**e**), 65% (**f**), 72% (**g**), and 77% (**h**) dropout rates. Each boxplot represents the result from 100 simulated datasets. *P* values are based on Student’s *t* test

We first evaluated the performance of SCRABBLE using simulated data where the ground truth is known. We used two simulation strategies. Strategy 1 is based on the Splatter method and generates completely synthetic data (Fig. 2a, Additional file 1: Figure S1). Splatter captures many features observed in the scRNA-seq data, including zero-inflation, gene-wise dispersion, and differing sequencing depths between cells [9]. Strategy 2 uses down-sampled real bulk RNA-seq dataset [10] (Fig. 3a, Additional file 1: Figure S3). Here, we introduced dropout events using an exponential function to control dropout rate (parameter *λ*) and a Bernoulli process to introduce dropout events at the corresponding dropout rate [4, 11] (see the “Methods” section). Using the 2 strategies, we simulated data with dropout rates corresponding to 60 to 87% zeros in the data. Moreover, to evaluate the robustness of imputation methods, at a given dropout rate, we simulated 100 data sets. It is well known that real RNA-seq data tend to have a characteristic property of inverse relationship between mean and variance [12]. We confirmed that our simulated data also contains this property using the mean-variance plot (Additional file 1: Figures S1 and S3).

To evaluate the performance of each method, we define the imputation error as the *L*_2_ norm of the difference between the imputed and the true data matrices. Using both types of simulated data across a range of dropout rates, we found that SCRABBLE outperforms four state-of-the-art methods (DrImpute, scImpute, MAGIC, and VIPER) (Figs. 2d–f and 3e–h). More importantly, the performance gain is observed across the full spectrum of gene expression levels (Figs. 2c and 3d, Additional file 1: Figures S2, S4-S6). All other methods led to imputed values that were significantly lower than the true values for > 88% (Fig. 2c) and > 40% (Fig. 3d) of the genes. In contrast, SCRABBLE led to imputed values that were significantly higher than the true values for 1% (Fig. 2c) and 2% (Fig. 3d) of the genes. The imputed data by SCRABBLE also captures the data substructure (i.e., clusters) better as embedded in the true data (Figs. 2b and 3c, Additional file 1: Figures S2, S4-S6).

**Fig. 4:**
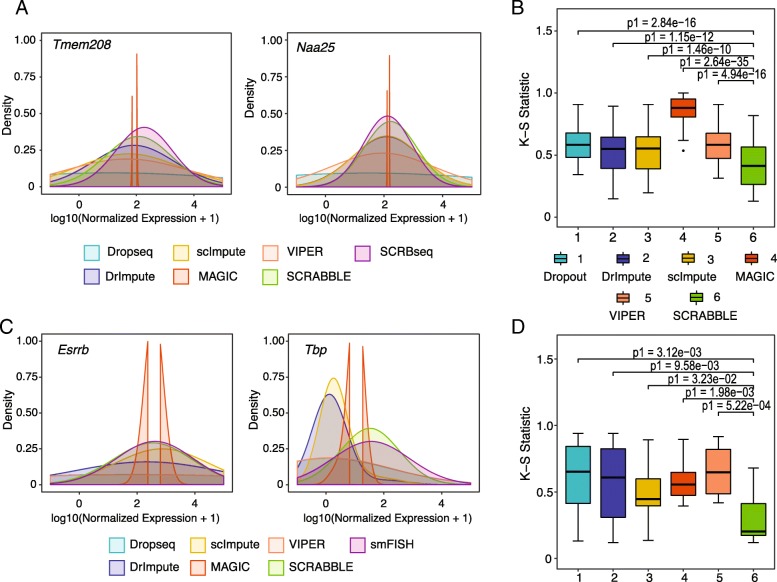
SCRABBLE-imputed gene expression distribution has a better match with gold standards. **a** Gene expression distributions of two representative genes in true (SCRB-Seq), dropout (Drop-Seq), and imputed data. **b** Boxplots of the agreement of gene expression distribution between true data (SCRB-Seq) and imputed data using Drop-Seq data as input to the methods. Agreement between the two distributions is measured using the Kolmogorov-Smirnov (KS) test statistic. A set of 56 genes in mouse ES cells is examined. **c** Gene expression distributions of two representative genes in smRNA FISH data and imputed data. **d** Boxplots of the agreement of gene expression distribution between smRNA FISH data and imputed data. *P* values are based on Student’s *t* test

Besides simulating dropout events, we also used a real scRNA-seq dataset [13] (and matched bulk RNA-seq [14]) for mouse embryonic stem cells (J1 line) where dropout events are identified by comparing the data generated using the Drop-Seq [15] and the SCRB-Seq [16] protocols. At the same sequencing depth, the former protocol has a higher dropout rate [13]. We identified 56 genes that have zero expression in at least 29% of the cells in the Drop-Seq data but non-zero expression levels in all cells in the SCRB-Seq data. We therefore used the expression levels of these 56 genes in the SCRB-Seq data as the gold standard and imputed the Drop-Seq data. We found that SCRABBLE achieves the best performance among all methods in terms of matching the distribution of gene expression between the imputed and gold-standard data (Fig. 4b, Additional file 2: Figure S7). The similarity between distributions is measured using the Kolmogorov-Smirnov test statistic. Like the performance using simulated data, the performance gain by SCRABBLE is observed across the full range of gene expression levels (Additional file 2: Figure S8). Figure 4a shows raw and imputed expression levels of two representative genes, *Tmem208* and *Naa25* (the rest of the genes are shown in Additional file 2: Figure S7). We observed the same performance gain by SCRABBLE in another set of 17 genes with dropout events in at least 39% of the cells (i.e., higher dropout rate, Additional file 2: Figure S9).

We further assess the performance of SCRABBLE using single-molecule RNA fluorescence in situ hybridization (smRNA FISH) data and scRNA-seq data measured on the same cell type, mouse embryonic stem cell line, E14 [17, 18]. We compared the distributions of the imputed expression and smRNA FISH measurements for the same set of 12 genes across single cells. Overall, the distributions of expression values imputed by SCRABBLE have the highest agreement with the smRNA FISH data (Fig. 4d), suggesting best performance by SCRABBLE. Figure 4c shows raw and imputed expression levels of two representative genes, *Esrrb* and *Tbp* (the rest of the genes are shown in Additional file 2: Figure S10).

**Fig. 5:**
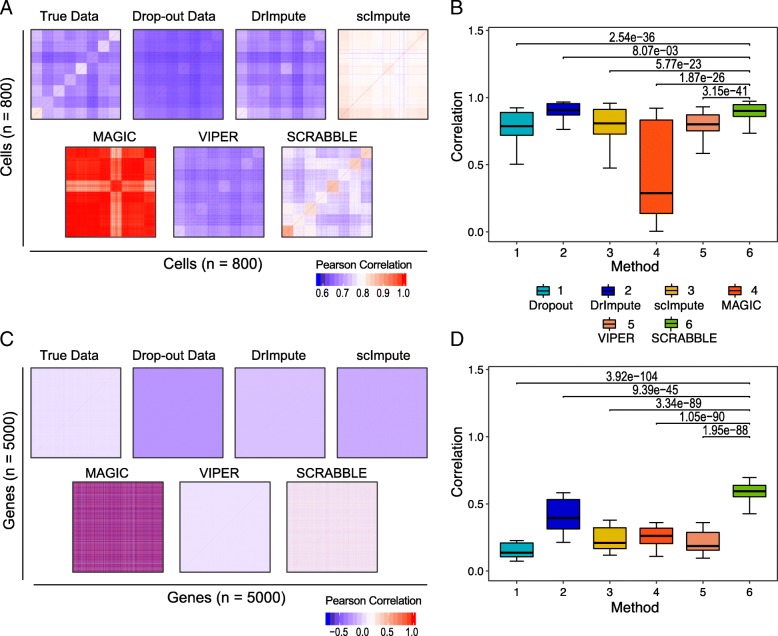
SCRABBLE better preserves the true cell-cell and gene-gene relationships in the data. **a** Representative cell-cell correlation matrices using true, dropout, and imputed data. The dropout rate is 72%. Values are Pearson correlation coefficients. **b** Pearson correlation between the cell-cell correlation matrices based on true and dropout/imputed data. Boxplots represent 100 sets of simulated data. *P* values are based on Student’s *t* test. **c** Representative gene-gene correlation matrices using true, dropout, and imputed data. **d** Pearson correlation between the gene-gene correlation matrices based on true and dropout/imputed data

A major application of scRNA-seq is to better understand the gene-gene and cell-cell relationships in a complex tissue. Thus, a good imputation method should preserve the data structure that reflects the true gene-gene and cell-cell relationships. We computed the gene-gene and cell-cell correlation matrices using the data simulated using strategy 2. Using Pearson correlation, we then determined the similarity between the correlation matrices based on true data and dropout/imputed data. Data imputed by SCRABBLE gave rise to a significantly higher correlation to the true cell-cell correlations than those imputed by the other four methods (Fig. 5b). Figure 5a shows a set of representative cell-cell correlation matrices based on true, dropout, and imputed data. As can be seen, SCRABBLE does the best job in capturing the true cell-cell correlation patterns among the four methods. MAGIC reports a large number of high correlations. However, most of those are false positives judging by the true cell-cell correlation matrix. This is because MAGIC tends to impute counts that are not affected by dropout and thus tends to flatten the data distribution towards the sample mean. Histograms of the correlation values are shown in Additional file 2: Figure S11. We note that all imputation methods tend to distort the true data distribution as suggested by the inflated correlations based on the imputed data (Additional file 2: Figure S11). Nevertheless, the higher agreement of cell-cell correlations using true data and SCRABBLE imputed data is observed using the data simulated with both strategies and across a range of dropout rates (Additional file 2: Figures S12 and S13).

For the gene-gene relationship, among the entire set of 5000 genes, data imputed by SCRABBLE results in the highest agreement with the gene-gene correlation pattern based on the true data (Fig. 5c, d). This higher agreement of gene-gene correlations is observed using the data simulated with both strategies and across a range of dropout rates (Additional file 2: Figures S14 and S15). Histograms of the correlation values are shown in Additional file 2: Figure S16.

The imputation procedure could inadvertently distort the clustering result. To evaluate this issue, we next computed the cell-cell and gene-gene correlations using cells/genes stratified based on their cluster membership (for cell-cell correlation) and on whether they are marker genes of a cluster (for gene-gene correlation). For cell-cell correlation, we computed the within- and between-cluster correlations across cells. For gene-gene correlation, we computed the correlations among marker genes and among marker and non-marker genes for a given cluster. For both cell-cell and gene-gene correlations, the distance between the two correlation distributions was quantified using the Kolmogorov-Smirnov (KS) statistic. Finally, the distortion of the clustering result is measured by comparing the KS statistic based on true data and imputed data. For both cell-cell (Additional file 2: Figures S17 and S18) and gene-gene (Additional file 2: Figures S19 and S20) correlations, SCRABBLE gives the smallest distortion compared to the other methods. The same performance gain is observed using the data simulated with strategy 1 (Additional file 2: Figures S21 and S22).

**Fig. 6:**
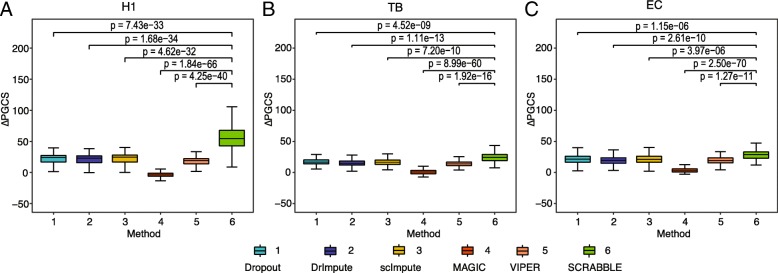
Pairwise expression correlation among pathway genes is improved using imputed data. A pathway gene correlation score (PGCS) measures the increase in expression correlation among pathway genes compared to a set of randomly selected genes of the same size. ΔPGCS is the difference in PGCS between imputed data and un-imputed data. For each data set (dropout or imputed data), a ΔPGCS value is computed for each pathway. Boxplot represents ΔPGCS values for 186 pathways in the IPA database. *P* value is based on Student’s *t* test. **a** Human h1 ES cells data (H1). **b** Human trophoblast (TB)-like cells data. **c** Human foreskin fibroblast cells (HFF)

Another way to evaluate the preservation of gene-gene relationship in the sample is by using pathway annotations because genes in the same pathway tend to have correlated expression. We applied SCRABBLE to matched the scRNA-seq and bulk RNA-seq data for seven cell types [19], H1 and H9 (human embryonic stem cell lines), human foreskin fibroblast (HFF), definitive endoderm cells (DEC), endothelial cells (EC), trophoblast (TB)-like cells, and neuronal progenitor cells (NPC). We defined a pathway gene correlation score (PGCS) which measures the increase in the expression correlation among the pathway genes compared to a set of randomly selected genes of the same size. We then computed the difference in PGCS (ΔPGCS) between the imputed data and un-imputed data. For a better imputation method, we expect to see a larger ΔPGCS value. Using pathway annotations from three databases, Ingenuity Pathway Analysis (IPA) [20], Kyoto Encyclopedia of Genes and Genomes (KEGG) [21], and REACTOME [22], we found SCRABBLE consistently produces larger ΔPGCS values compared to the other four methods (Fig. 6, Additional file 2: Figures S23-S25) in all cell types examined, suggesting data imputed by SCRABBLE better preserves the gene-gene relationship information in the data.

**Fig. 7:**
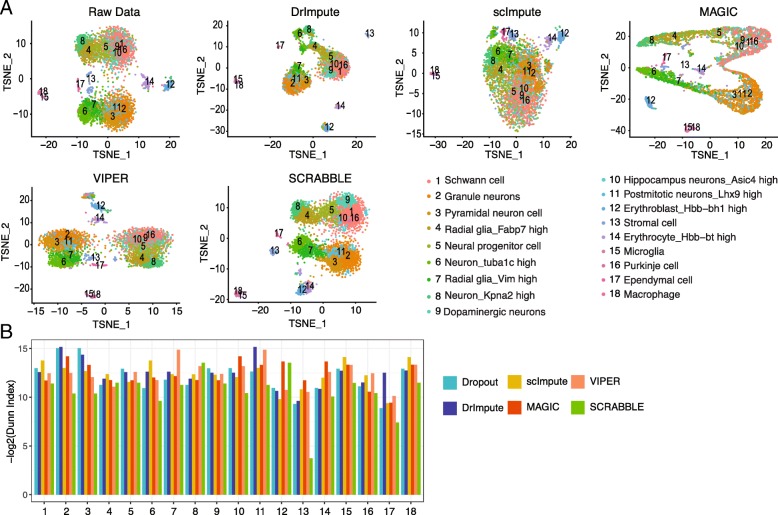
SCRABBLE improves the clustering analysis. **a** Clustering results using un-imputed and imputed data by various methods. scRNA-seq data was clustered using *K*-nearest neighbor clustering and visualized using t-SNE. The number of clusters (*K*) was based on the ones provided by the authors. Cell type of each cluster was identified based on marker genes provided by the authors. **b** Quantification of cluster quality using the Dunn index

To demonstrate that SCRABBLE can improve the downstream analysis, we applied it to the matched scRNA-seq [23] and bulk RNA-seq [24] of 8 mouse tissues, including fetal brain (4369 cells), fetal liver (2699 cells), kidney (4673 cells), liver (4685 cells), lung (6940 cells), placenta (4346 cells), small intestine (6684 cells), and spleen (1970 cells). Using both raw and imputed scRNA-seq data, multiple cell types (as determined by signature gene expression) can be detected using *K*-nearest neighbor clustering (Fig. 7a, Additional file 2: Figures S26-S32). This result further demonstrates that SCRABBLE can capture cell heterogeneity in complex tissues although it uses average gene expression values of the bulk data. To evaluate the clustering quality using either raw or imputed data, we used the Dunn index which computes the ratio of minimal inter-cluster distance versus maximal intra-cluster distance. A higher Dunn index indicates a better separation among clusters. We found that the use of imputed data by SCRABBLE results in improved clustering quality as compared to clustering without imputation and with imputed data by the other four methods (Fig. 7b, Additional file 2: Figures S26-S32).

SCRABBLE has three parameters (i.e., *α*, *β*, and *γ*). To evaluate the robustness of SCRABBLE over parameter setting, we varied the values of the three parameters by 0.1-, 0.5-, 2-, and 10-folds and performed imputation using data simulated using strategy 1 with the dropout rate of 83%. We found that the median percentage change in imputed data before and after changing the parameter is less than 5% for both *α* and *β* and less than 15% for *γ* (Additional file 2: Figure S33), suggesting SCRABBLE is very robust with regard to parameter setting. The sets of SCRABBLE parameters used in this study are provided in Additional file 3: Table S2. We also benchmarked the running time of SCRABBLE. The higher imputation accuracy of SCRABBLE comes with a price of slower running time. For dataset containing fewer than 2000 cells, SCRABBLE has a better or comparable speed as that of VIPER (Additional file 2: Figure S34). As the dataset size exceeds 5000 cells, SCRABBLE is twice as slow as VIPER, mostly due to the computationally expensive process of iterative single value decomposition.

## Discussion

SCRABBLE addresses several deficiencies of existing methods. First, several methods impute dropout events by using cell-cell distance, as quantified by either Euclidean distance or kernel distance. Such distance measures may not reflect the true relationship among cells. SCRABBLE relies on the framework of matrix regularization which does not use cell-cell distance measure. Second, SCRABBLE borrows information from bulk RNA-seq data to impute dropout data in order to reduce unwanted bias during imputation. Finally, since we transform the mathematical model of SCRABBLE to a convex optimization problem, the existence of the optimal solution is guaranteed mathematically. Our comprehensive analysis using both simulated and real experimental data suggests that SCRABBLE achieves significant improvement in terms of recovering dropout events and preserving cell-cell and gene-gene relationships in the samples. As an example of SCRABBLE’s utility to facilitate downstream analysis, we show that using SCRABBLE-imputed data leads to a better clustering quality and helps identify different cell types in complex tissues.

One caveat about our method is the use of average values of bulk RNA-seq data. It may reduce the ability of the method to capture biological heterogeneity in the data. However, we believe the advantage of using bulk data outweighs the disadvantage. Additionally, the other two terms of our model, projection and low rank, enable SCRABBLE to detect heterogeneity and covariation.

As other types of single-cell omics data become more abundant, such as single-cell DNA methylation and ATAC data, our method provides a general framework for imputing and integrating these data for new discoveries.

## Conclusions

Here, we describe the SCRABBLE algorithm and software package. SCRABBLE imputes single-cell RNA-seq data by using bulk RNA-seq data both as a constraint and as prior information. We show leveraging information in bulk RNA-seq data significantly improves the quality of imputed data. With SCRABBLE, existing or newly generated bulk RNA-seq data can be used to increase the utility of single-cell RNA-seq data.

## Methods

### The mathematical model of SCRABBLE

The input to SCRABBLE includes the scRNA-seq and bulk RNA-seq data on consistent cells/tissues. A matrix, *X*_0_, represents expression values from scRNA-seq data with columns representing *m* genes and rows representing *n* cells. A vector, *D*, represents the average expression levels of all genes in the bulk RNA-seq data across *N* samples.

The output matrix $\overset{\wedge }{X}$ of SCRABBLE is the imputed matrix with the same dimensions as the input matrix *X*_0_. The algorithm is based on the following mathematical model:

1 $$ \overset{\wedge }{X}=\underset{X\ge 0}{\mathrm{argmin}}\left(\frac{1}{2}{\left\Vert {P}_{\varOmega }(X)-{X}_0\right\Vert}_F^2+\alpha \mathrm{Rank}(X)+\beta {\left\Vert aX-D\right\Vert}_2^2\right) $$

where *P*_*Ω*_(·) is the projection operator that forces *x*_*ij*_ to be zeros (*x*_*ij*_ is the element at the *i*th row and the *j*th column of the matrix *X* and (*i*, *j*) ∉ Ω); otherwise, the value of *x*_*ij*_ is kept as it is. *Ω* is determined by *X*_0_ and (*i*, *j*) ∈ Ω if ${x}_{ij}^0\ne 0$, where ${x}_{ij}^0$ is the element at the *i*th row and the *j*th column of the matrix *X*_0_. Rank(*X*) is the rank of the matrix *X*. *a* is a row vector with the size 1 by *n* and each element in *a* is $\frac{1}{n}$. *α* and *β* are the parameters of the mathematical model. *α* is the weight for the rank of the imputed data matrix. Large *α* results in reduced heterogeneity across the cells. *β* is the weight for the agreement between the aggregated scRNA-seq and bulk RNA-seq data. *β* is proportional to *α* and the size of the imputed data matrix.

### Iterative optimization of the objective function during imputation

Since the objective function in Eq. (1) is not convex due to the rank function, the relaxed form of the objective function is employed to compute the optimal solution as follows.

2 $$ \overset{\wedge }{X}=\underset{X\ge 0}{\mathrm{argmin}}\left(\frac{1}{2}{\left\Vert {P}_{\varOmega }(X)-{X}_0\right\Vert}_F^2+\alpha {\left|\left|X\right|\right|}_{\ast }+\beta {\left\Vert aX-D\right\Vert}_2^2\right) $$

where ||∙||_∗_ is the nuclear norm, which is the convex envelope of the rank function. We use the following three steps to calculate $\overset{\wedge }{X}$.

Step 1: Convert the original optimization problem into a convex optimization problem with a linear constraint by introducing the auxiliary variable *Y.*

3 $$ \overset{\wedge }{\Big(X,}\overset{\wedge }{Y}\Big)=\mathrm{argmin}\left(\frac{1}{2}{\left\Vert {P}_{\varOmega }(X)-{X}_0\right\Vert}_F^2+\alpha {\left|\left|Y\right|\right|}_{\ast }+\beta {\left\Vert aX-D\right\Vert}_2^2+{\chi}_{X\ge 0}\right) $$

such that *X* − *Y* = 0.

where *χ*_*X* ≥ 0_ is the characteristic function which takes the value of 0 if *X* ≥ 0 and ∞ otherwise.

Step 2: Convert the constrained optimization problem to the unconstrained optimization problem using the augmented Lagrangian method and solve the unconstrained optimization problem using the alternating direction method of multipliers (ADMM) [25].

4 $$ \left(\overset{\wedge }{X,}\overset{\wedge }{Y}\right)=\underset{X\ge 0}{\mathrm{argmin}}\left(\frac{1}{2}{\left\Vert {P}_{\varOmega }(X)-{X}_0\right\Vert}_F^2+\alpha {\left|\left|Y\right|\right|}_{\ast }+\beta {\left\Vert aX-D\right\Vert}_2^2+{\chi}_{X\ge 0}+<\Lambda, X-Y{>}_F+\frac{\gamma }{2}{\left|\left|X-Y\right|\right|}_F^2\right) $$

The ADMM iteration scheme can be written as follows:

5 $$ {X}^{k+1}=\mathrm{argmin}\left(\frac{1}{2}{\left\Vert {P}_{\varOmega }(X)-{X}_0\right\Vert}_F^2+\beta {\left\Vert aX-D\right\Vert}_2^2+{\chi}_{X\ge 0}+<{\Lambda}^k,X-{Y}^k{>}_F+\frac{\gamma }{2}{\left\Vert X-{Y}^k\right\Vert}_F^2\right) $$

6 $$ {Y}^{k+1}=\mathrm{argmin}\left(\alpha {\left|\left|Y\right|\right|}_{\ast }+<{\Lambda}^k,{X}^{k+1}-Y{>}_F+\frac{\gamma }{2}{\left|\left|{X}^{k+1}-Y\right|\right|}_F^2\right) $$

7 $$ {\Lambda}^{k+1}={\Lambda}^k+\gamma \left({X}^{k+1}-{Y}^{k+1}\right) $$

We take the derivative with respect to *X* to obtain the iteration scheme of Eq. (5).

$$ \left({P}_{\varOmega }(X)-{X}_0\right)+\beta {a}^T\left( aX-D\right)+{\Lambda}^k+\gamma \left(X-{Y}^k\right)=0 $$

$$ {P}_{\varOmega }(X)+\left(\beta {a}^Ta+\gamma I\right)X=\gamma {Y}^k+\beta {a}^TD+{X}_0-{\Lambda}^k $$

Let *βa*^*T*^*a* + *γI* = *W* and *βa*^*T*^*D* + *X*_0_ = *T*

8 $$ {P}_{\varOmega }(X)+ WX=\gamma {Y}^k+T-{\Lambda}^k $$

Then, we rewrite Eq. (6) as:

9 $$ {\displaystyle \begin{array}{c}{Y}^{k+1}=\mathrm{argmin}\ \alpha {\left\Vert Y\right\Vert}_{\ast }+<{\Lambda}^k,{X}^{k+1}-Y{>}_F+\frac{\gamma }{2}{\left\Vert {X}^{k+1}-Y\right\Vert}_F^2\\ {}=\mathrm{argmin}\frac{\alpha }{\gamma }{\left\Vert Y\right\Vert}_{\ast }+<\frac{\Lambda^k}{\gamma },{X}^{k+1}-Y{>}_F+\frac{1}{2}{\left\Vert {X}^{k+1}-Y\right\Vert}_F^2+\frac{1}{2}{\left\Vert \frac{\Lambda^k}{\gamma}\right\Vert}_F^2\\ {}=\mathrm{argmin}\frac{\alpha }{\gamma }{\left\Vert Y\right\Vert}_{\ast }+\frac{1}{2}{\left\Vert \frac{\Lambda^k}{\gamma }+{X}^{k+1}-Y\right\Vert}_F^2\end{array}} $$

Step 3: Based on Eqs. (7), (8), and (9), we could get the following iteration schemes.

10 $$ {x}_{ij}=\left\{\begin{array}{c}{\left(\frac{\gamma {y}_{ij}^k+{t}_{ij}-{\Lambda}_{ij}^k-\sum \limits_{i=1,j\ne i}^n{w}_{ij}{x}_{ij}}{w_{ii}}\right)}_{+}\kern2.75em \left(i,j\right)\notin \varOmega \\ {}{\left(\frac{\gamma {y}_{ij}^k+{t}_{ij}-{\Lambda}_{ij}^k-\sum \limits_{i=1,j\ne i}^n{w}_{ij}{x}_{ij}}{w_{ii}+1}\right)}_{+}\kern2.5em \left(i,j\right)\in \varOmega \end{array}\right. $$

11 $$ {\displaystyle \begin{array}{l}{Y}^{k+1}=\mathrm{SVT}\left(\frac{X^{k+1}+{\Lambda}^k}{\gamma },\frac{\alpha }{\gamma}\right)\\ {}{\Lambda}^{k+1}={\Lambda}^k+\gamma \left({X}^{k+1}-{Y}^{k+1}\right)\end{array}} $$

where Eqs. (10) and (11) are the iteration schemes for Eqs. (5) and (6), represents the singular value thresholding algorithm [26] defined for any matrix *Z* and *τ* > 0 as follows:

$$ \mathrm{SVT}\left(Z,\tau \right)=U\operatorname{diag}\left\{\left({\sigma}_i-\tau \right)\right\}{V}^T $$

Here, *Z* = *U* diag({*σ*_*i*_}_1 ≤ *i* ≤ *r*_)*V*^*T*^ is the singular value decomposition of *Z*, and *σ*_*i*_s are the positive singular values. Λ^*k*^, *X*^*k*^, and *Y*^*k*^ are the *k*th iteration matrix of Λ, *X*, and Y, respectively. In addition, *x*_*ij*_, ${y}_{ij}^k$, ${\Lambda}_{ij}^k$, *w*_*ij*_, and *t*_*ij*_ are the elements at the *i*th row and *j*th column in the matrices *X*, *Y*^*k*^, Λ^*k*^, *W*, and *T*, respectively. The convergence of ADMM for convex optimization problems has been extensively studied in the literature [25, 27]. Since the objective function in (2) is convex and non-negative, the problem has at least one global solution. This global structure of the objective function in Eq. (2) allows the above algorithm to converge more quickly compared to other evolutionary algorithms [28]. The penalty parameter *γ* plays an important role in solving the objective function in Eq. (9) using the singular value thresholding algorithm combined with the parameter *α*. Overall, *α*, *β*, and *γ* are the three necessary parameters of SCRABBLE.

### Generation of simulated data

We simulated the scRNA-seq data consisting of three cell types using the Bioconductor package Splatter (version 1.4.3) [9]. We used the *splatSimulateGroup* function to generate the simulation data with 1000 cells and 800 genes. Three clusters were embedded in each simulated dataset. The size of each cluster was controlled by the parameter “group.prob” to be 0.2, 0.35, and 0.45. The parameter controlling the probability that a gene is differentially expressed in each group was set equal to 0.045. The location parameter and the scale factor parameter of randomly generating multiplication factors from a log-normal distribution were set to be 0.1 and 0.4, respectively. Dropout midpoints (parameter “dropout_mid” in Splatter) were used to control the dropout rates in the simulated data. For instance, dropout midpoints of 4, 5, and 5.5 correspond to 71%, 83%, and 87% dropout rates in the simulated data, respectively. The corresponding bulk RNA-seq data were the mean values of genes in the true scRNA-seq data. The dropout RNA-seq and bulk RNA-seq data matrices are the inputs of the imputation methods. To determine the performance stability of the methods, we generated 100 datasets for each dropout midpoints.

### Generation of simulated data using bulk RNA-seq data

We used the bulk RNA-seq dataset of mouse hair follicles from [10]. In total, the dataset contains 20 different combinations of anatomic sites and developmental time points. We used the following procedures to generate the simulated datasets (Fig. 3a): (1) we randomly selected 8 out of the 20 conditions; (2) for each condition, we generated 100 resampled datasets. The means and standard deviations of genes were calculated for each condition based on the 100 resampled datasets; (3) 100 new datasets were generated based on the mean and the standard deviation of each gene; (4) in order to reduce the computation cost, we randomly selected 5000 genes from 20,721 genes in the above data matrices. The final data matrix represents 800 cells and 5000 genes; and (5) we made the dropout rate of each gene in each cell following an exponential function ${e}^{-\lambda \bullet \mathrm{mean}\_{\mathrm{expression}}^2}$ [4, 11], where *λ* determines the dropout rate of scRNA-seq data. Zero values are introduced into the simulated data for each gene in each cell based on the Bernoulli distribution defined by the corresponding dropout rate. The corresponding bulk RNA-seq data are the mean values of genes in the scRNA-seq data without dropouts. To determine the performance stability of the methods, we generated 100 datasets for each dropout rate.

### Running of other imputation methods

We benchmarked DrImpute, scImpute MAGIC, and VIPER packages in this manuscript. For DrImpute (version 1.0), we used the following default parameter settings described in the Quick Start section of the user manual: ks = 10:15, dists = c(“spearman,” “pearson”), fast = FALSE, dropout.probability.threshold = 0, n.dropout = 10,000, n.background = 10,000, and mc.cores = 1. For scImpute (version 0.0.9), we used the following default parameter setting described in the Quick Start section of the user manual: labeled = FALSE, drop_thre = 0.5, and Kcluster = 1 in all analysis. For MAGIC (version 1.3.0 implemented in Python), we used the following default parameter setting, *k* = 10, *a* = 15, *t* = “auto”, n_pca = 100, knn_dist = “euclidean”, n_jobs = 1, and random_state = none. For VIPER (version 0.1.1), we used the following parameter setting: num = 5000, percentage.cutoff = 0.1, minbool = FALSE, and alpha = 1.

## Additional files

Additional file 1: **Figures S1-S6.** Supplementary figures. (PDF 17836 kb)

Additional file 2: **Figures S7-S34.** Supplementary figures. (DOCX 9400 kb)

Additional file 3: **Tables S1 and S2.** Supplementary tables. (PDF 41 kb)
