# Supplementary material for: SCRABBLE: single-cell RNA-seq imputation constrained by bulk RNA-seq data
Source: Genome Biol. 2019 May 6;20:88. doi: 10.1186/s13059-019-1681-8 (PMC6501316; doi:10.1186/s13059-019-1681-8)

Supplementary Figures

Fig S1. Mean-variance plot of data simulated using the Splatter package (related to Figure 2). Each row shows the mean-variant plots of true and drop-out data simulated by the Splatter package, as well as imputed data by the different imputation methods. The dropout rates for each row are shown in the right most column.

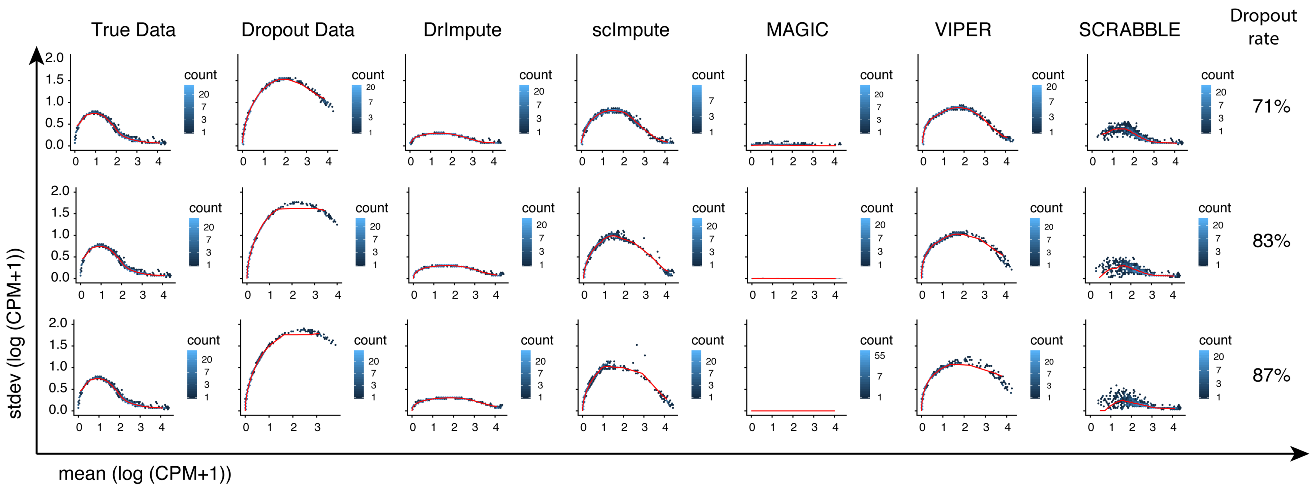

Fig S2. Performance using data simulated with the Splat method (related to Figure 2). (**A**,**B**) Performance on simulated data with 71% dropout rate. (**C**,**D**) Performance on simulated data with 87% dropout rate. (**A**,**C**), tSNE plots. (**B**,**D**) MA plots of true, dropout, and imputed data.

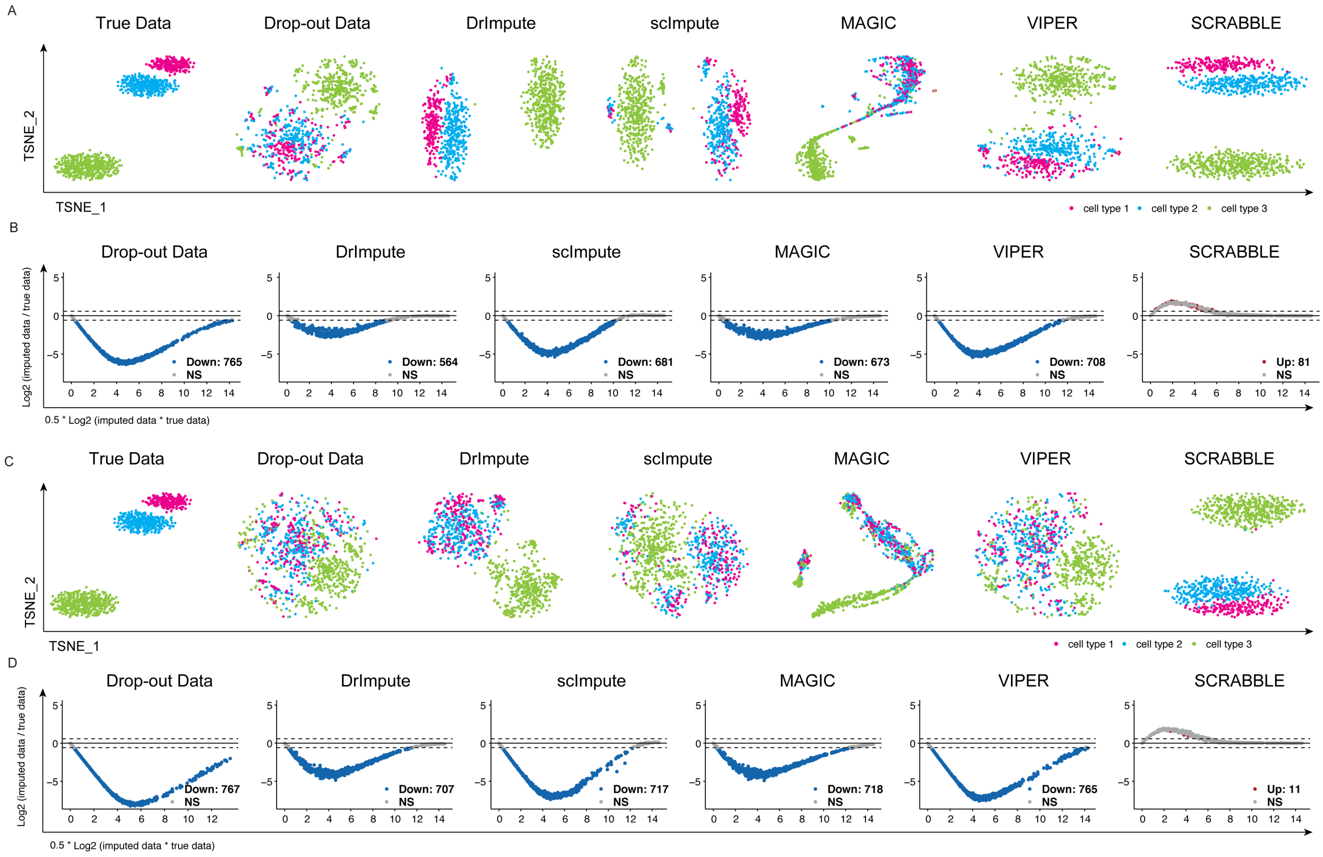

Fig S3. Mean-variance plot of data simulated using down-sampling of real RNA-Seq data (related to Figure 3). Each row shows the mean-variant plots of true and drop-out data, as well as imputed data by the different imputation methods. The dropout rates for each row are shown in the right most column.

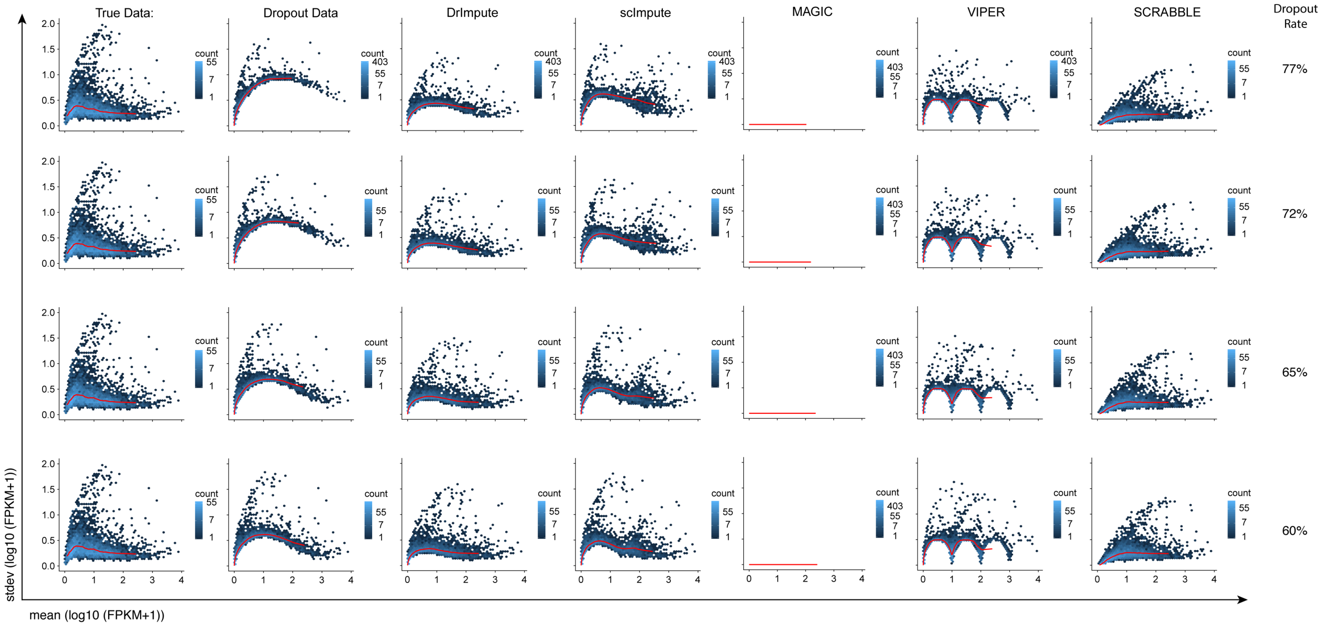

Fig S4. Performance using data simulated by down-sampling of real bulk RNA-Seq data and dropout rate of 60% (related to Figure 3). (**A**) Visualization of simulated true, droput and imputed data. (**B**) tSNE plots of true, dropout and imputed data. (**C**) MA plots of true, dropout, and imputed data. Significance of difference between true and imputed expression values was calculated using paired t-test. P-value < 0.05 was used as the significance threshold.

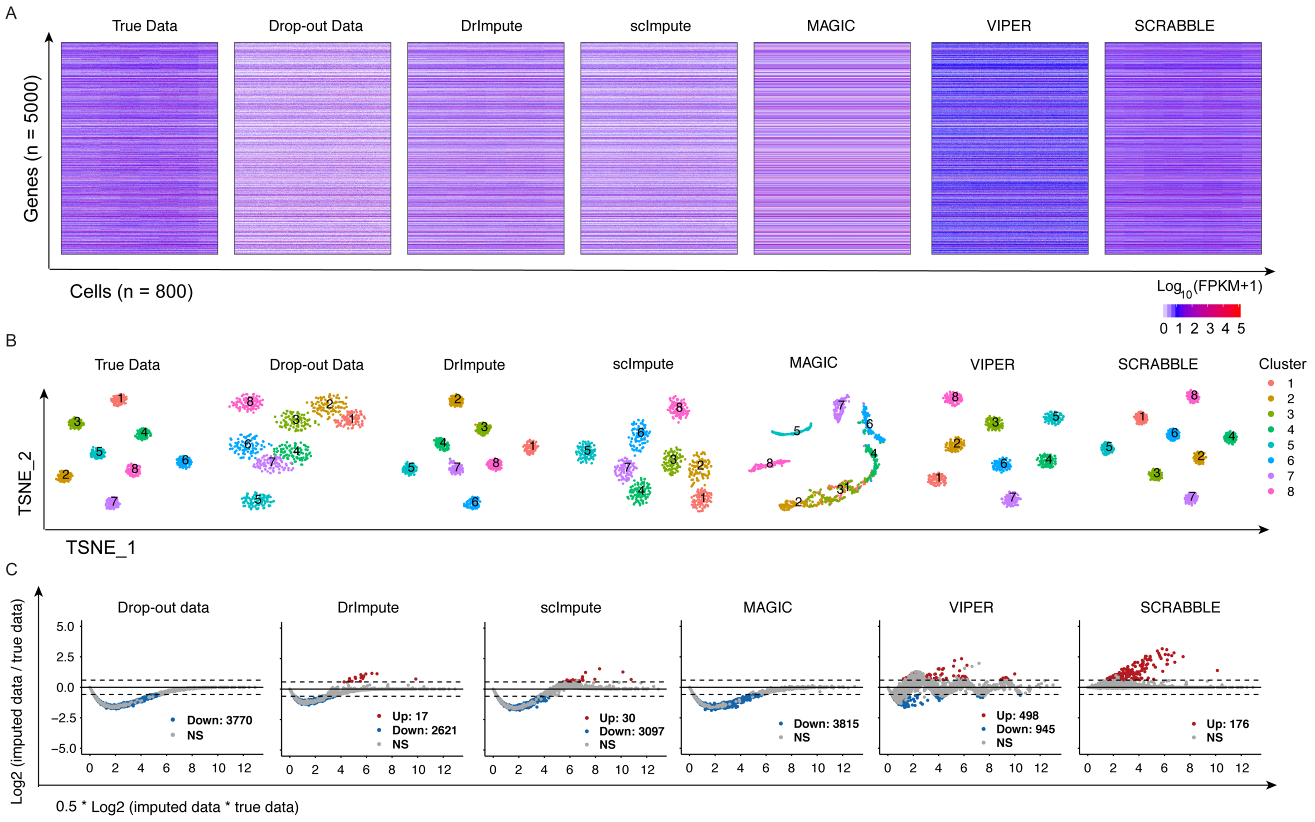

Fig S5. Performance using data simulated by down-sampling of real bulk RNA-Seq data and dropout rate of 65% (related to Figure 3). (**A**) Visualization of simulated true, dropout, and imputed data. (**B**) tSNE plots of true, dropout, and imputed data. (**C**) MA plots of true and dropout/imputed data. Significance of difference between true and imputed expression values was calculated using paired t-test. P-value < 0.05 was used as the significance threshold.

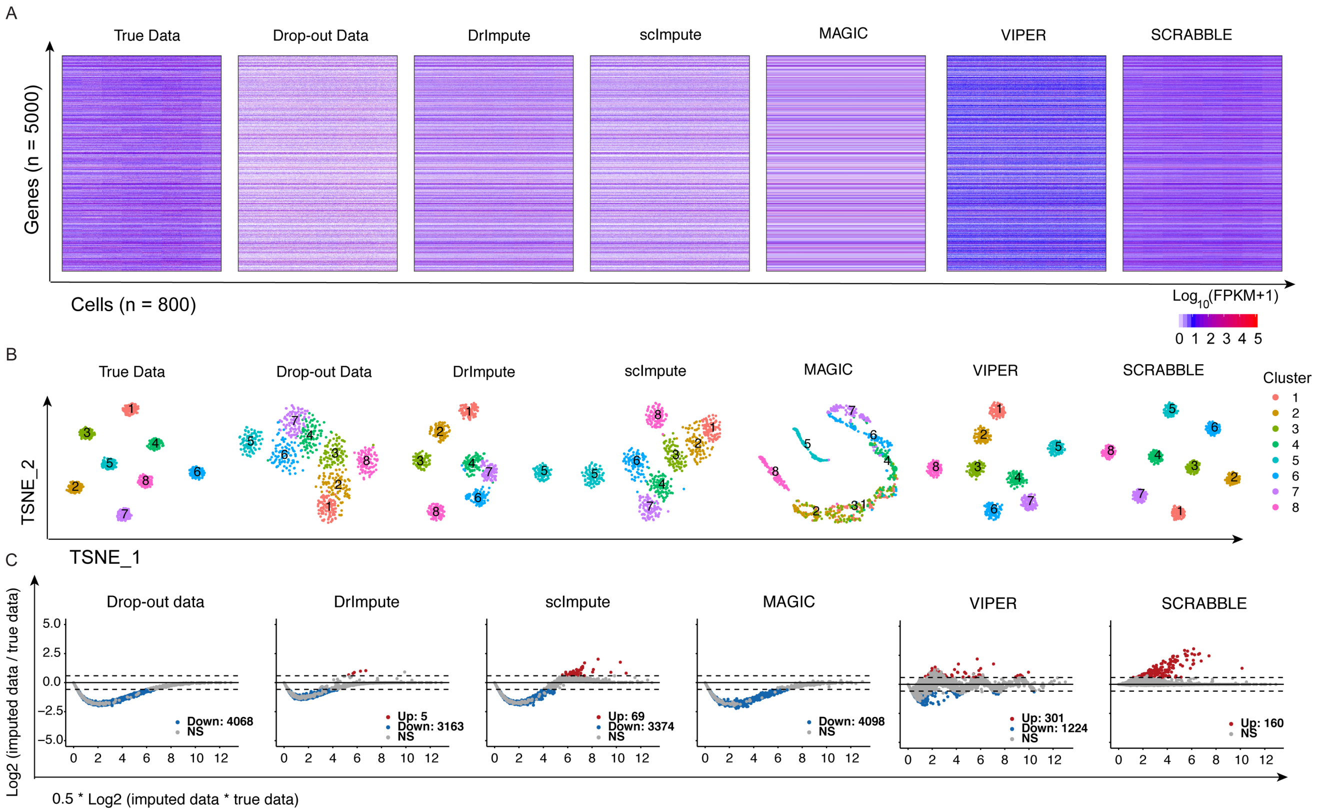

Fig S6. Performance using data simulated by down-sampling of real bulk RNA-Seq data and dropout rate is 77% (related to Figure 3). (**A**) Visualization of simulated true, dropout, and imputed data. (**B**) tSNE plots of true, dropout, and imputed data. (**C**) MA plots of true and dropout/imputed data. Significance of difference between true and imputed expression values was calculated using paired t-test. P-value < 0.05 was used as the significance threshold.

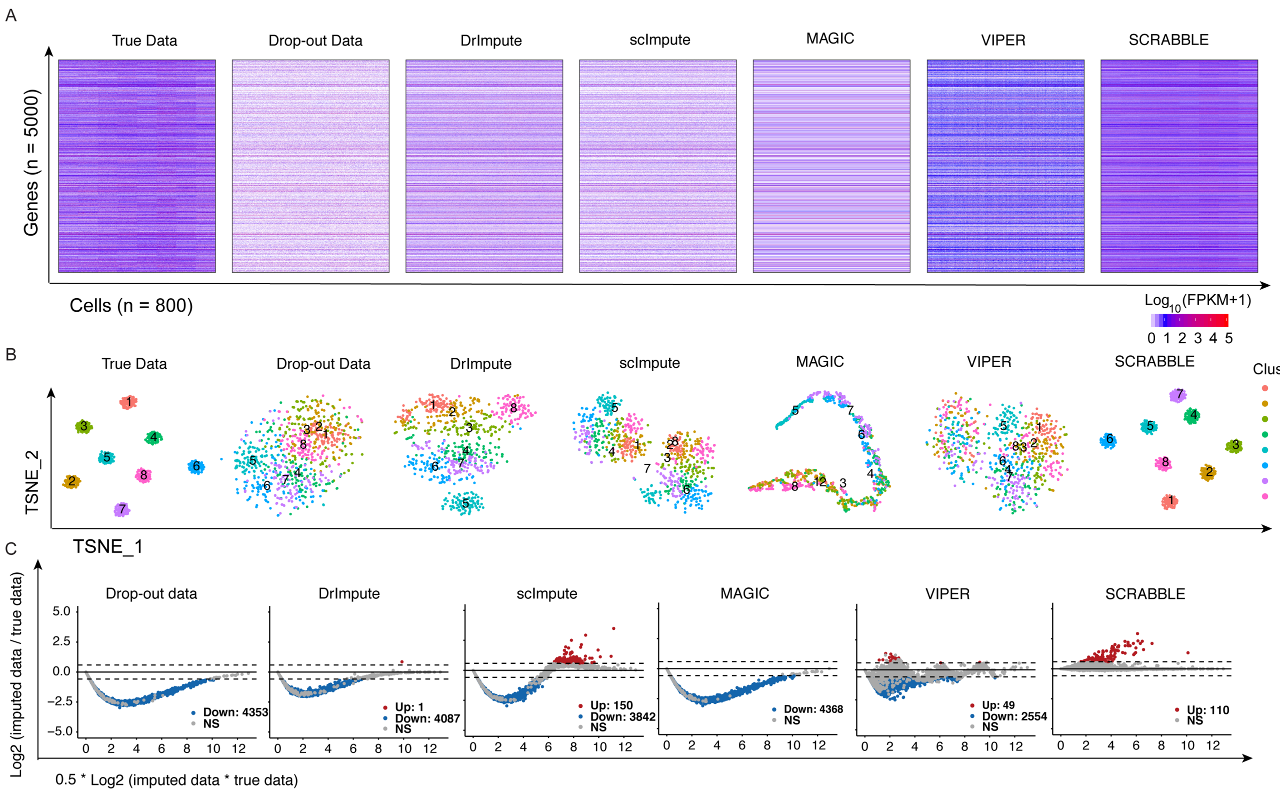

Supplement: Supplementary file 1 — Figures S1-S6. Supplementary figures. (PDF 17836 kb) [file 13059_2019_1681_MOESM1_ESM.pdf]
