# Supplementary material for: SCRABBLE: single-cell RNA-seq imputation constrained by bulk RNA-seq data
Source: Genome Biol. 2019 May 6;20:88. doi: 10.1186/s13059-019-1681-8 (PMC6501316; doi:10.1186/s13059-019-1681-8)

**Fig S7. Distribution of gene expression values in true, drop-out, and imputed scRNA-Seq data (related to Figure 4A) (related to Figure 4A).** Density plots for expression values of 56 genes are shown. These 56 genes have zero expression in at least 29% of the cells in the Drop-Seq data but nonzero expression levels in all cells in the SCRB-Seq data. Therefore, SCRB-Seq data is treated as true expression values. Drop-Seq data is treated as the drop-out data which are imputed using DrImpute, scImpute, MAGIC, and SCRABBLE.

**
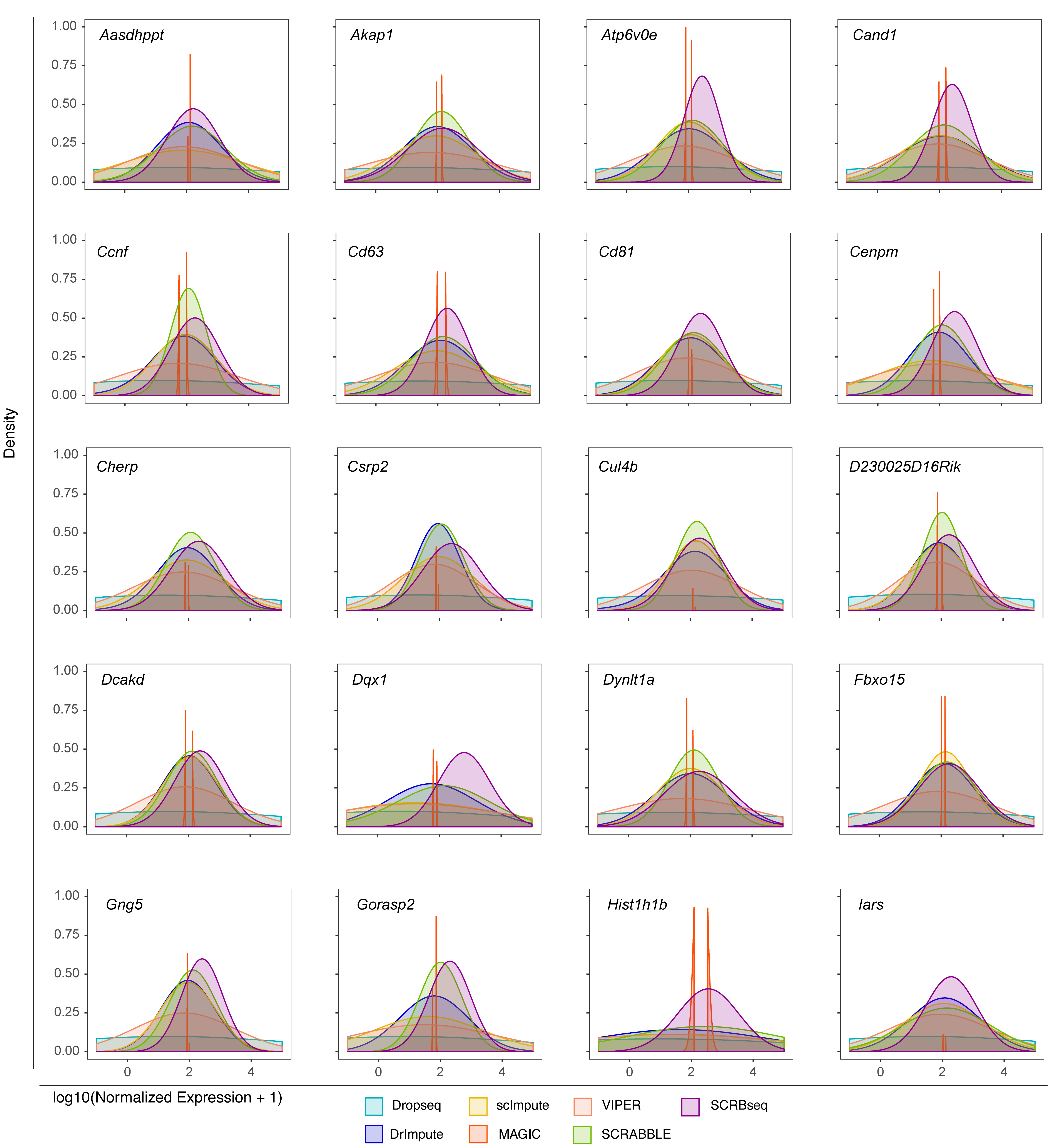
**

**
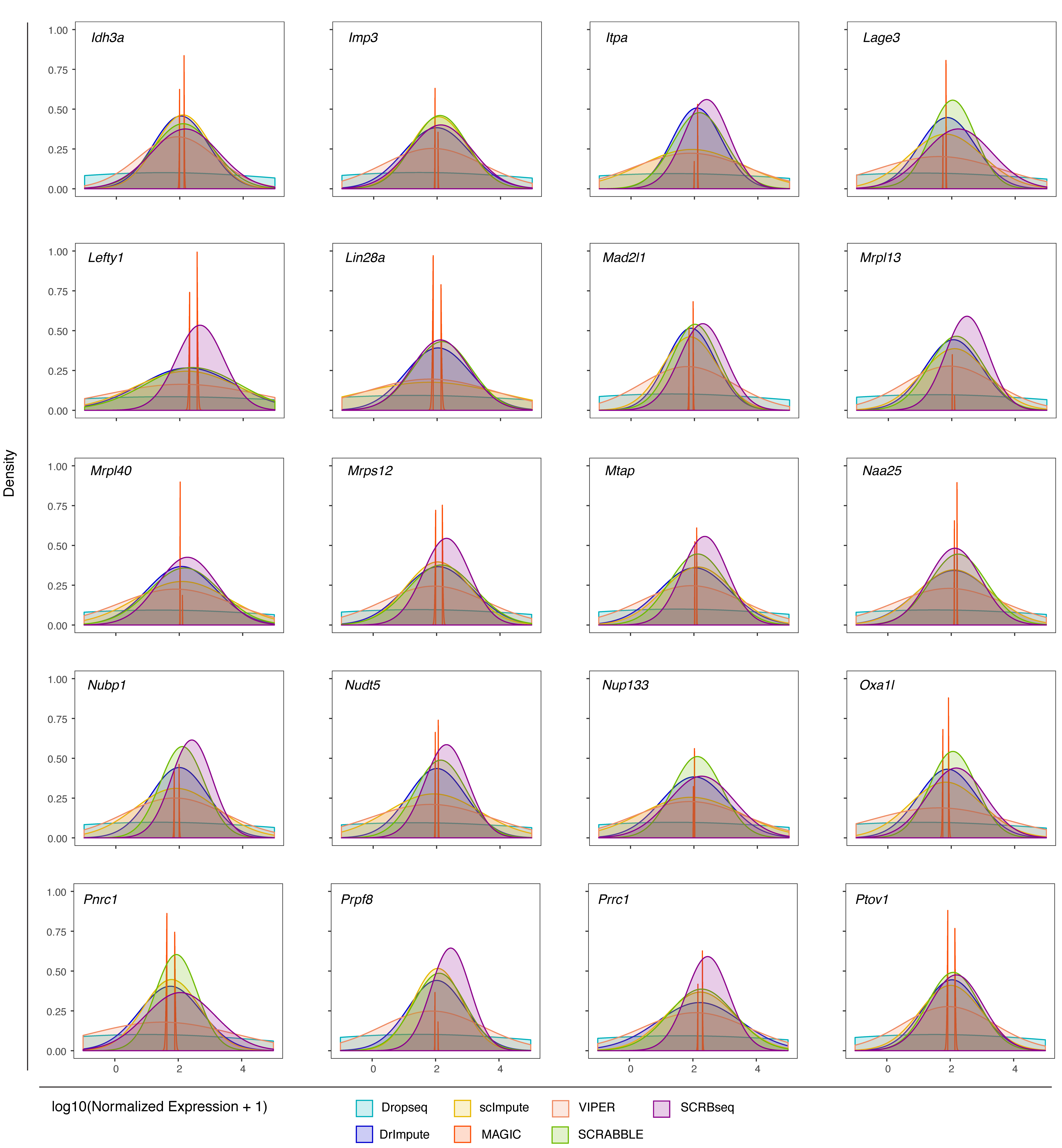
**

**
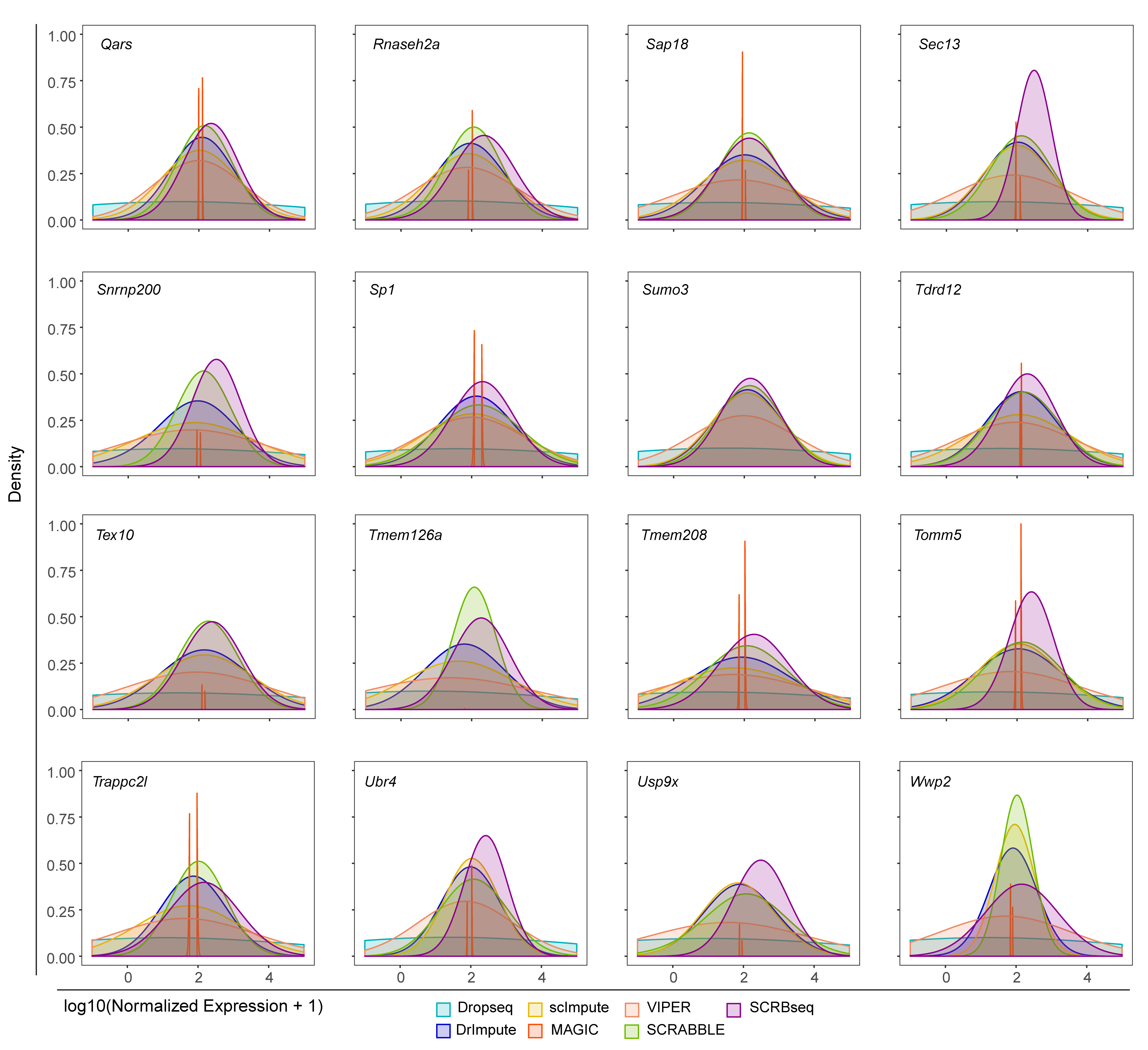
**

**Fig S8. KS statistic as a function of gene expression level (related to Figure 4).** Agreement of gene expression distributions between true data (SCRB-Seq) and imputed data using Drop-Seq data was measured using the Kolmogorov-Smirnov (KS) test statistic. Each vertical line represents a gene.

**
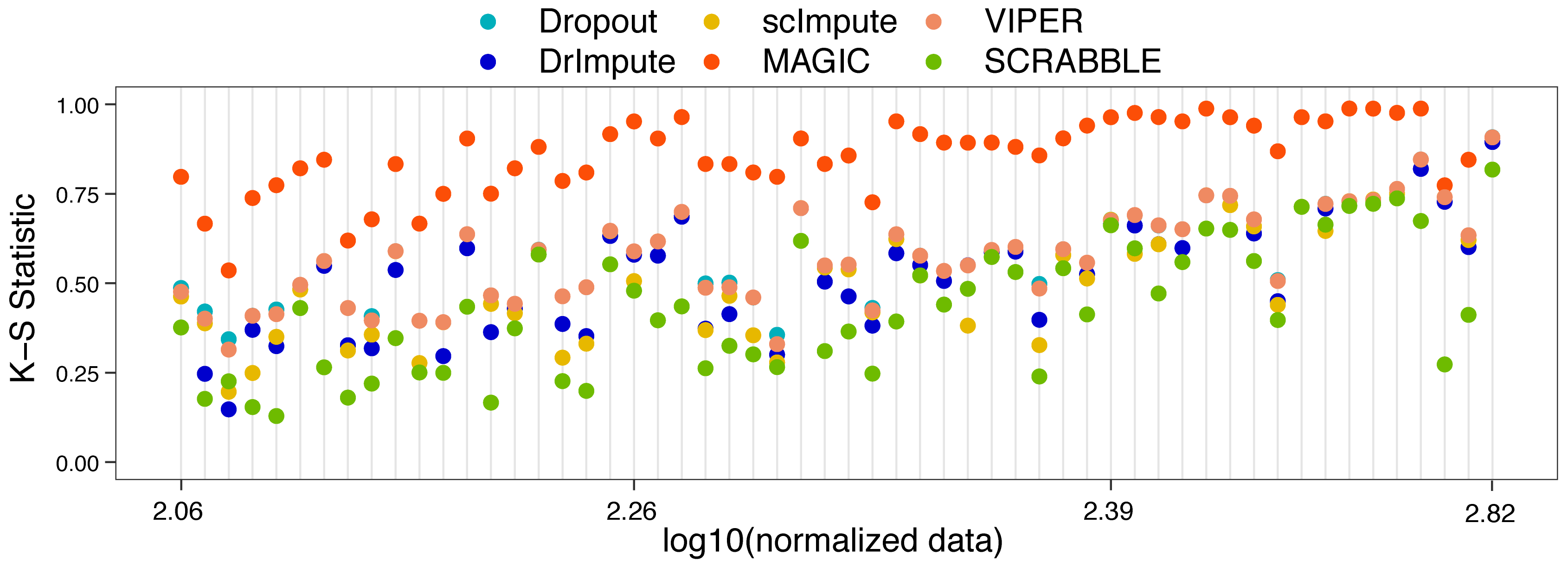
**

**Fig S9. Boxplot of the agreement of gene expression distribution between true data (SCRB-Seq) and imputed data using Drop-Seq data as input to the methods (related to Figure 4B).** A set of 17 genes in mouse ES cells with higher drop-out rate (compared to the 56 genes in Figure 4A) is examined. Agreement between two distributions is measured using the Kolmogorov-Smirnov (KS) test statistic. P-values are based on Student’s t-test.

**
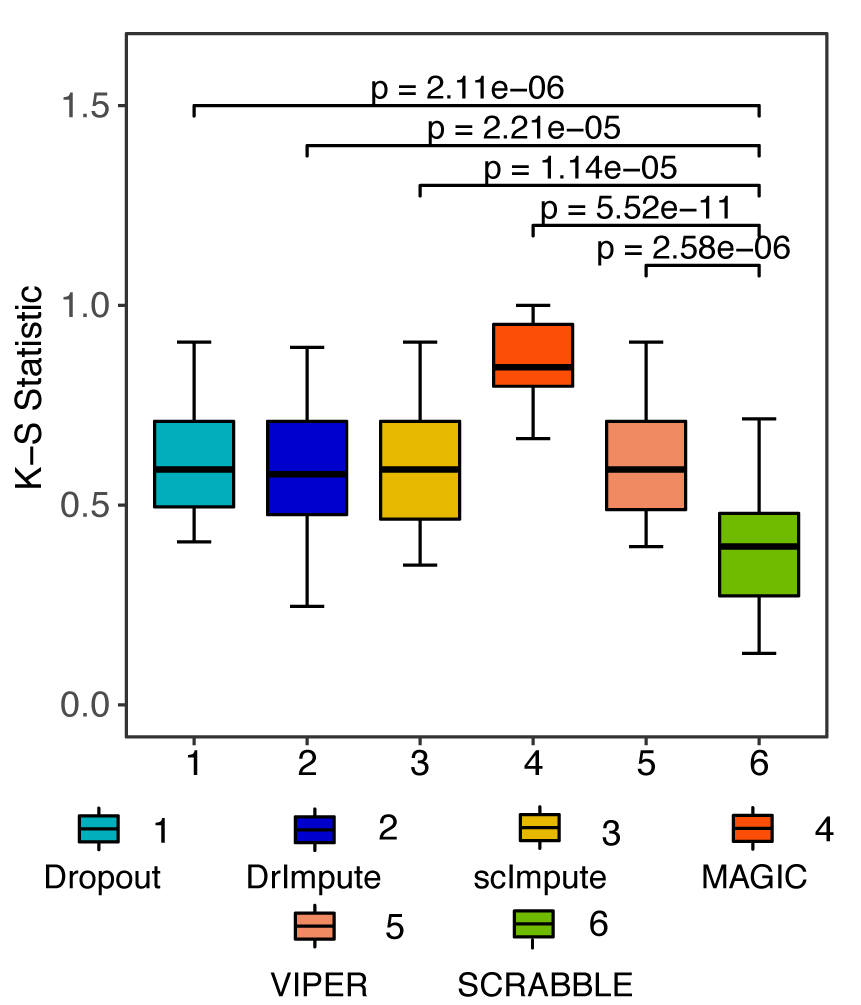
**

**Fig S10. Distribution of gene expression in imputed scRNA-Seq data and smRNA FISH data (related to Figure 4C).** Density plots for expression values of 12 genes are shown.

**
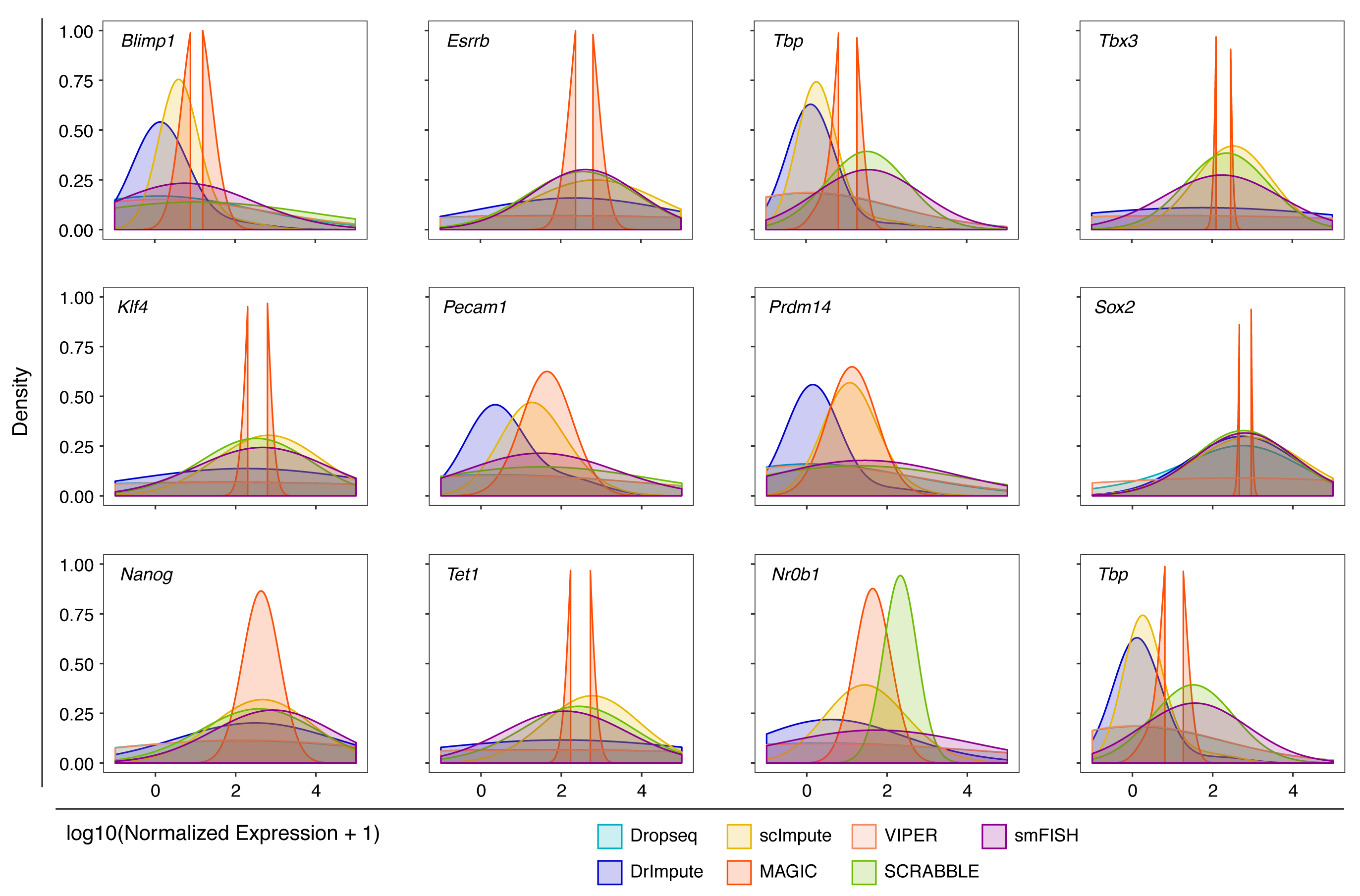
**

**Fig S11. Histograms of cell-cell correlation of simulated data using down-sampling of real bulk RNA-Seq data (related to Figure 5A)**. Each row shows histograms based on a given dropout rate.


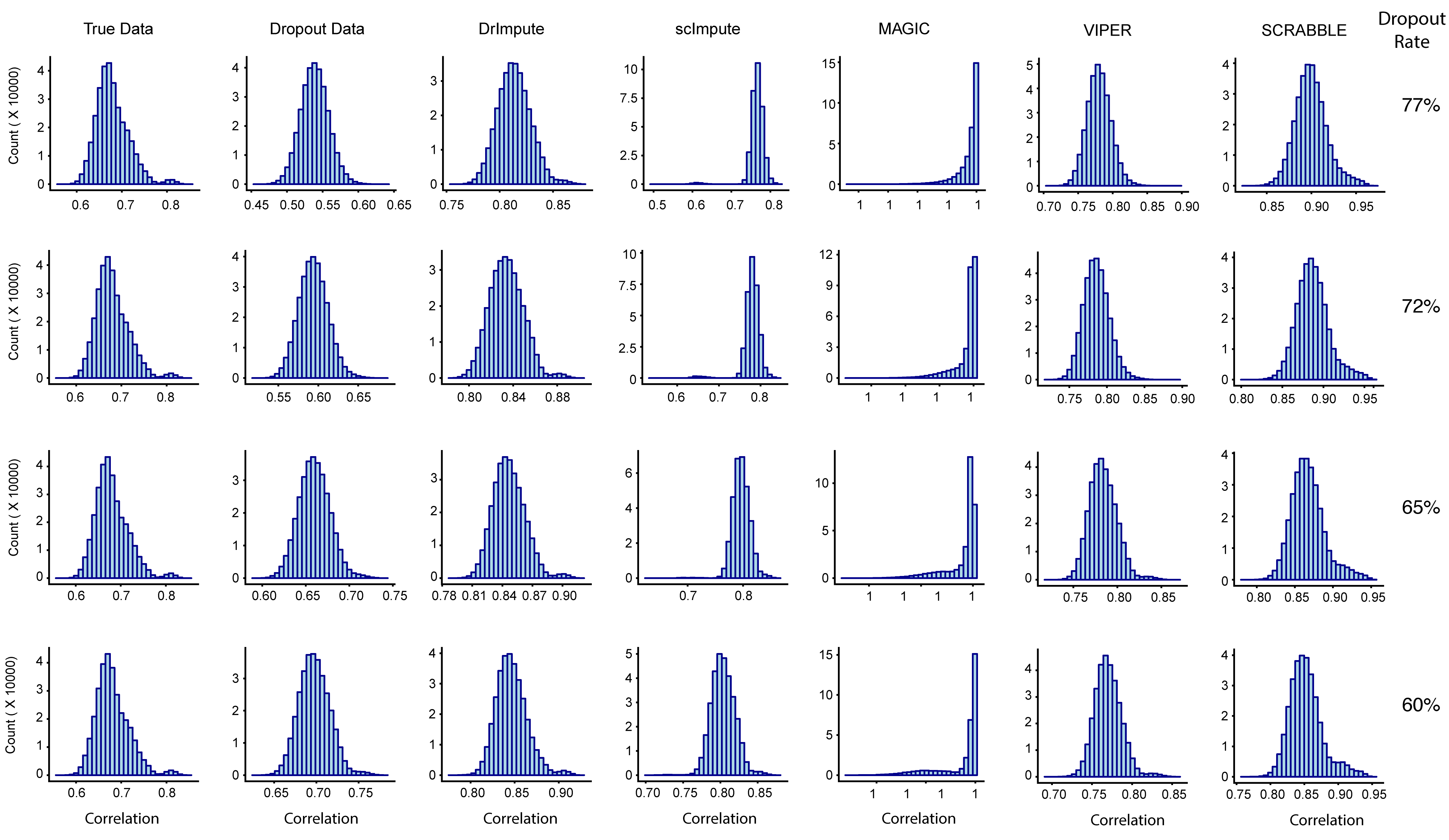


**Fig S12. Evaluation of cell-cell correlation using simulated data generated using simulation strategy 1 (related to Figure 5B).** Each boxplot of cell-cell correlation error is based on 100 sets of simulated data. Values in the boxplots are Pearson correlation between cell-cell correlation matrices based on true data and imputed data. P-values are based on Student’s t-test.

**
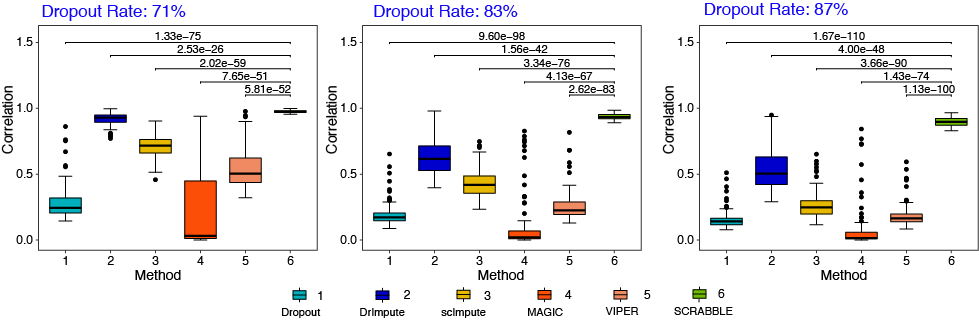
**

**Fig S13. Evaluation of cell-cell correlation using additional simulated data generated using simulation strategy 2 (related to Figure 5B).** In addition to the drop-out rate of 72% in Figure 5, data sets drop-out rates of 60%, 65%, and 77% were used. Each boxplot is based on 100 sets of simulated data. Values in the boxplots are Pearson correlation between cell-cell correlation matrices based on true data and imputed data. P-values are based on Student’s t-test.


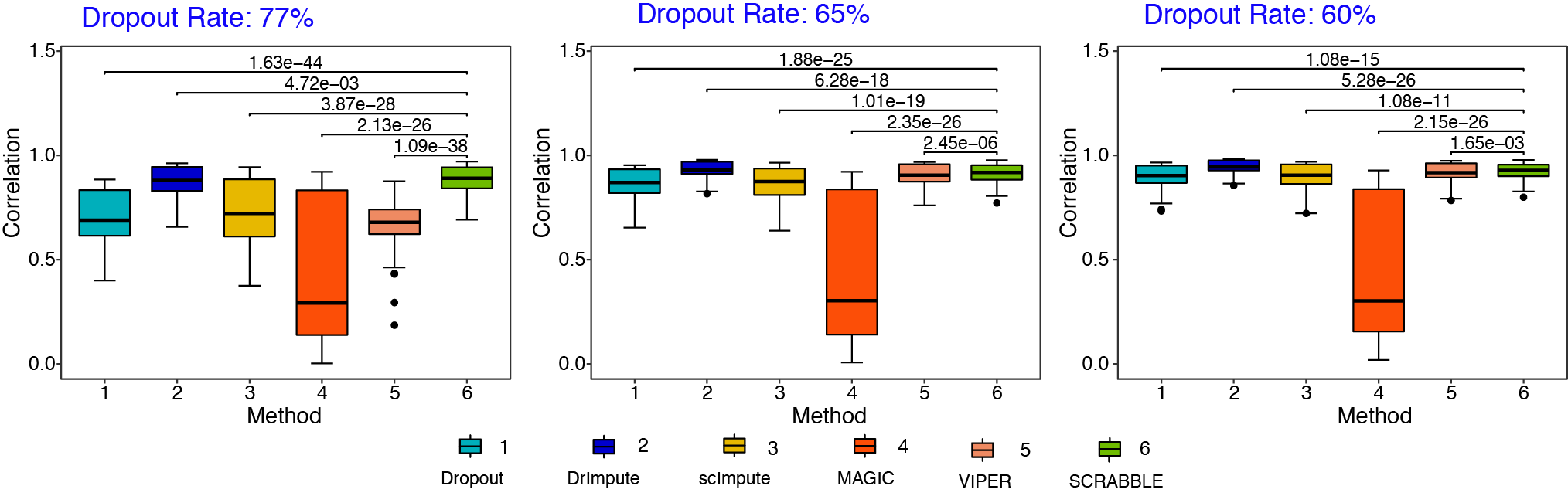


**Fig S14. Evaluation of gene-gene correlation using simulated data generated using simulation strategy 1 (related Figure 5D).** Each boxplot of gene-gene correlation error is based on 100 sets of simulated data. Values in the boxplots are Pearson correlation between cell-cell correlation matrices based on true data and imputed data. P-values are based on Student’s t-test.


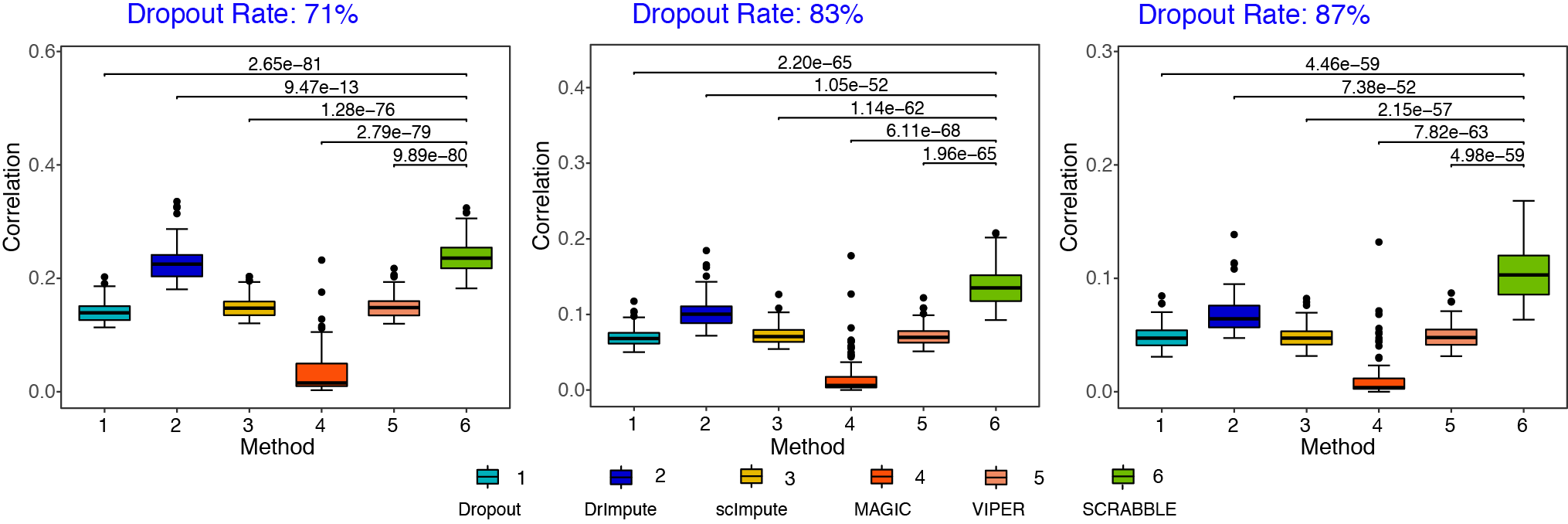


**Fig S15. Evaluation of gene-gene correlation using additional simulated data generated using simulation strategy 2 (related to Figure 5D).** In addition to the drop-out rate of 72% in Figure 5, data sets drop-out rates of 60%, 65%, and 77% were used. Each boxplot is based on 100 sets of simulated data. Values in the boxplots are Pearson correlation between cell-cell correlation matrices based on true data and imputed data. P-values are based on Student’s t-test.

**
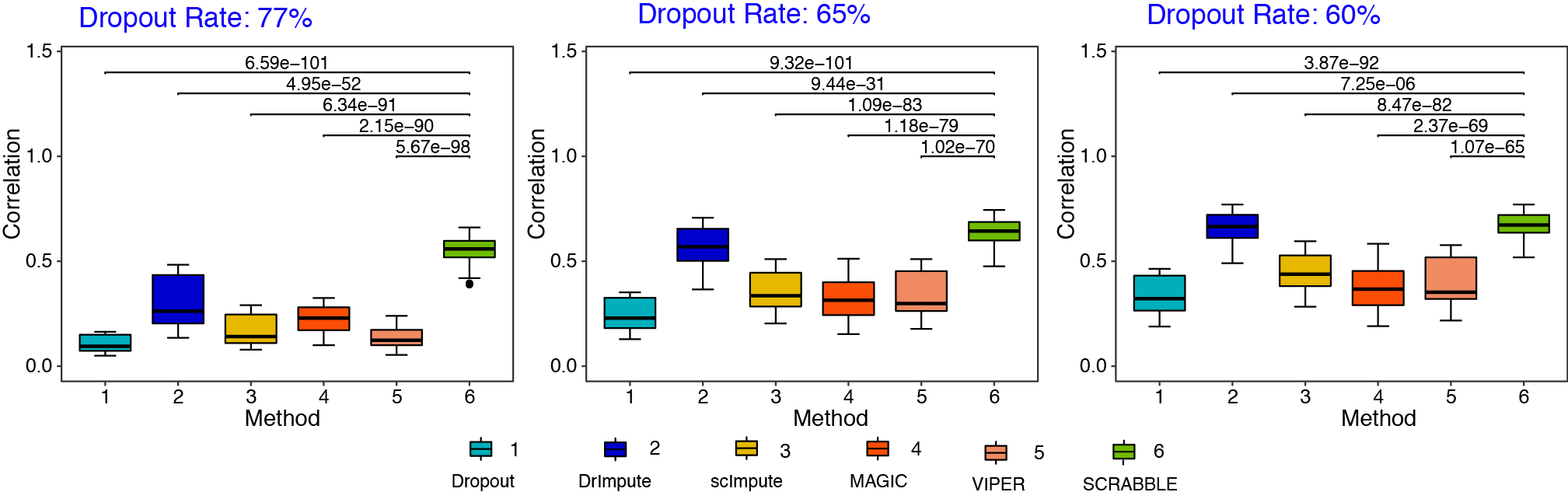
**

**Fig S16. Histograms of gene-gene correlation of simulated data using down-sampling of real bulk RNA-Seq data (related to Figure 5C)**. Each row shows histograms based on a given dropout rate.

**
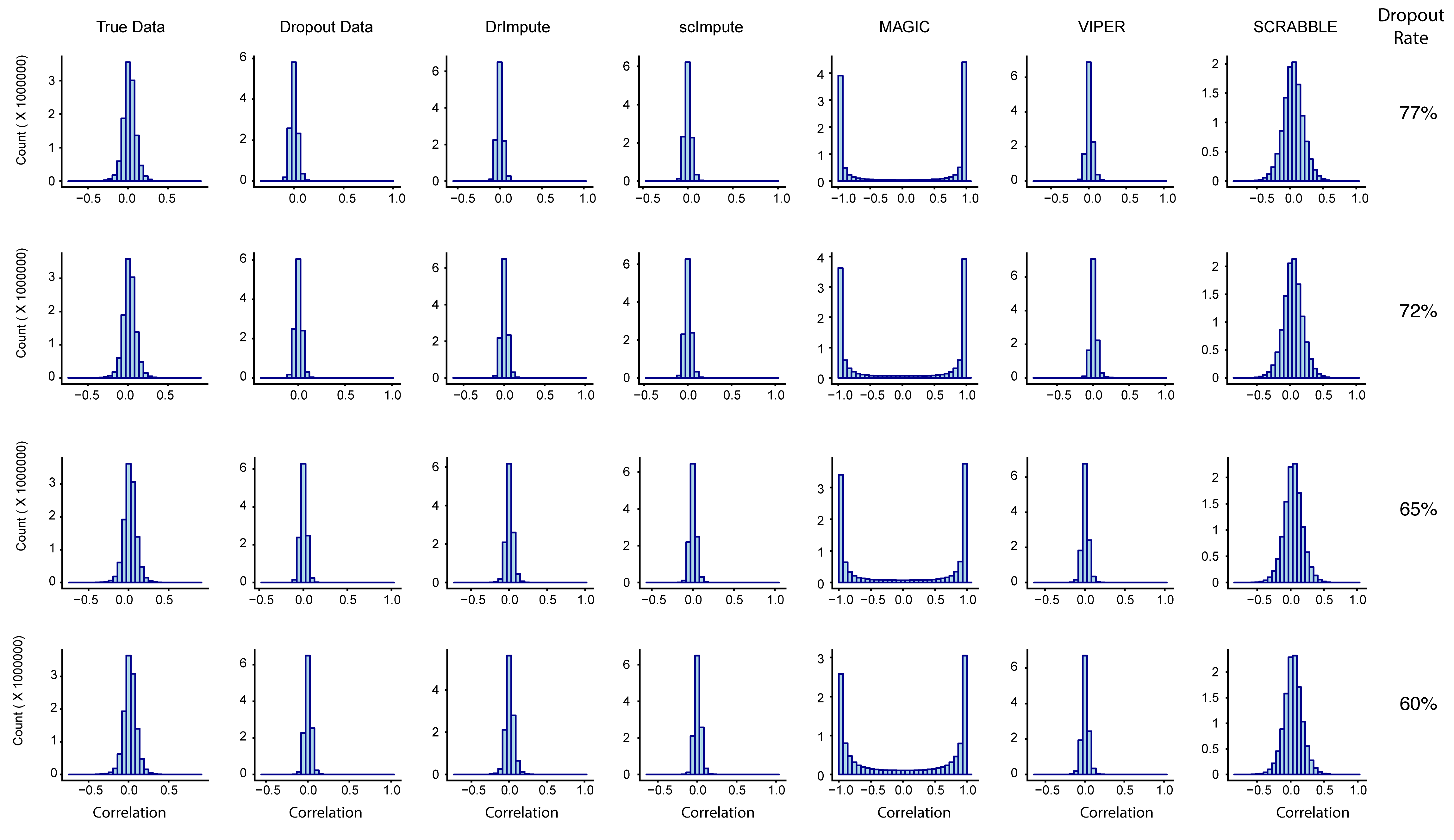
**

**Fig S17. Within and between cluster cell-cell correlations at the dropout rate of 72% (related to Figure 5A and 5B)**. Each row represents a cell cluster and shows the density plots of cell-cell correlation for cells within the cluster and cells between two clusters. KS statistic is used to quantify the distance between the within and between-cluster correlation distributions.


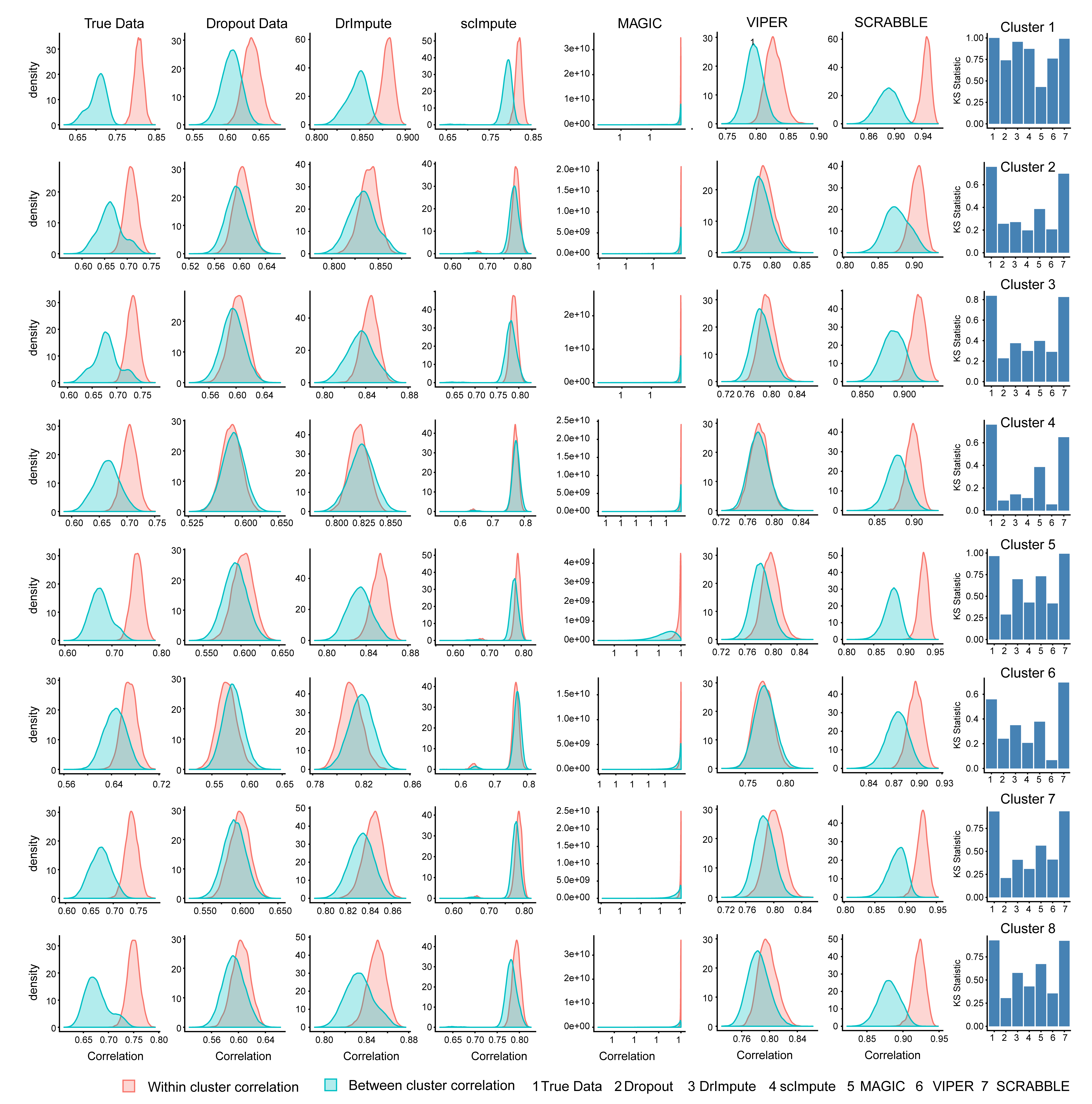


**Fig S18. Distance between within and between cluster cell-cell correlations using data generated with simulation strategy 2 (related to Figure 5A and 5B)**. Distance between within and between cluster correlation is quantified using KS statistic. Values in each boxplot are the differences in the KS statistic between imputed and true data. Each boxplot is based on 100 sets of simulated data. P-values are based on Student’s t-test.

**
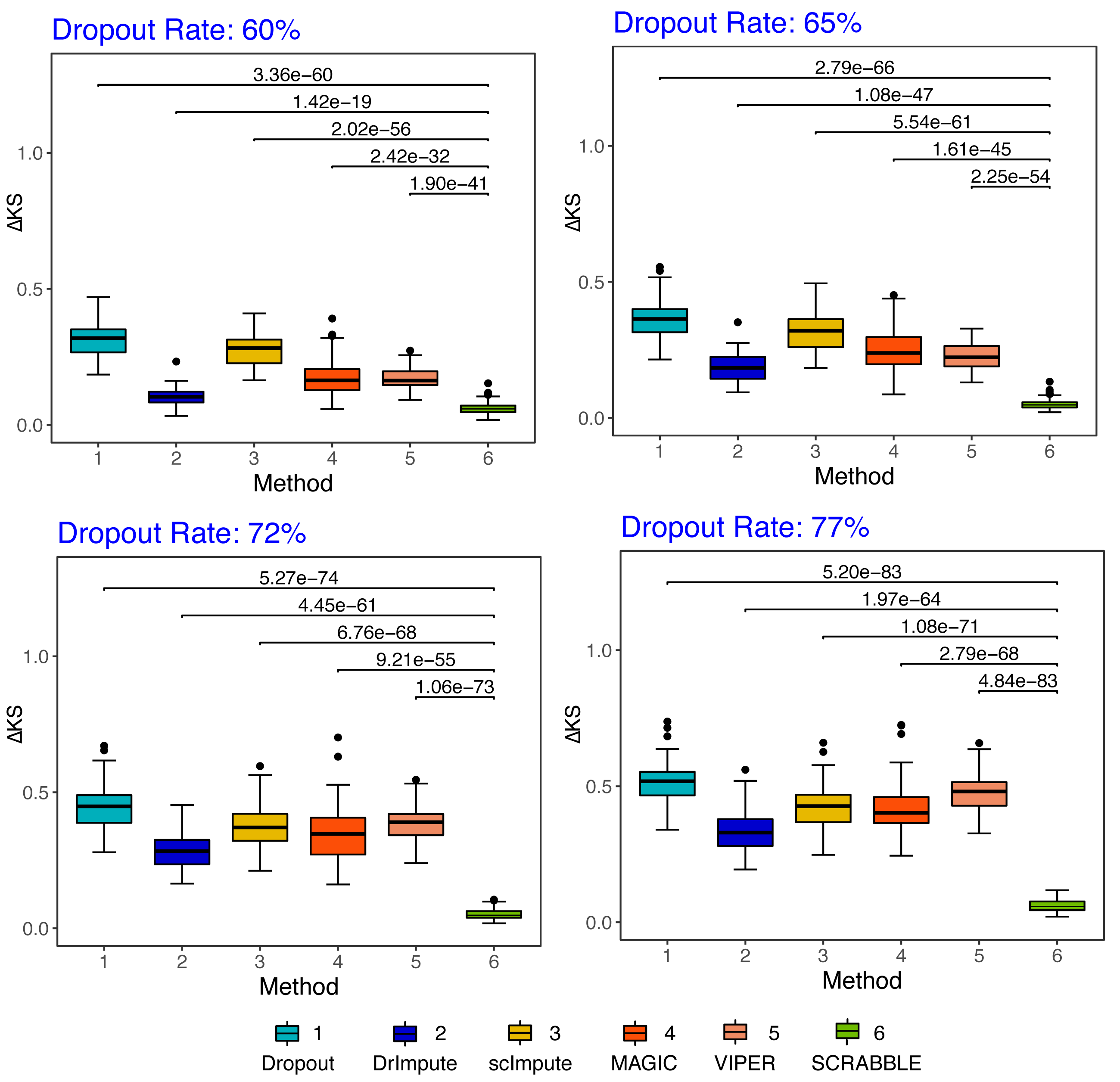
**

**Fig S19. Gene-gene correlations stratified based on marker genes using data with a dropout rate of 72% (related to Figure 5C and 5D)**. Each row represents a cell cluster and shows the density plots of gene-gene correlation among marker genes and among marker genes and non-marker genes for the given cluster. KS statistic is used to quantify the distance between the two correlation distributions. Marker genes for each cluster is defined using the SC3 method (version 1.10.1), using the default AUC threshold of 0.85 and p-value threshold of 0.01 for each gene.


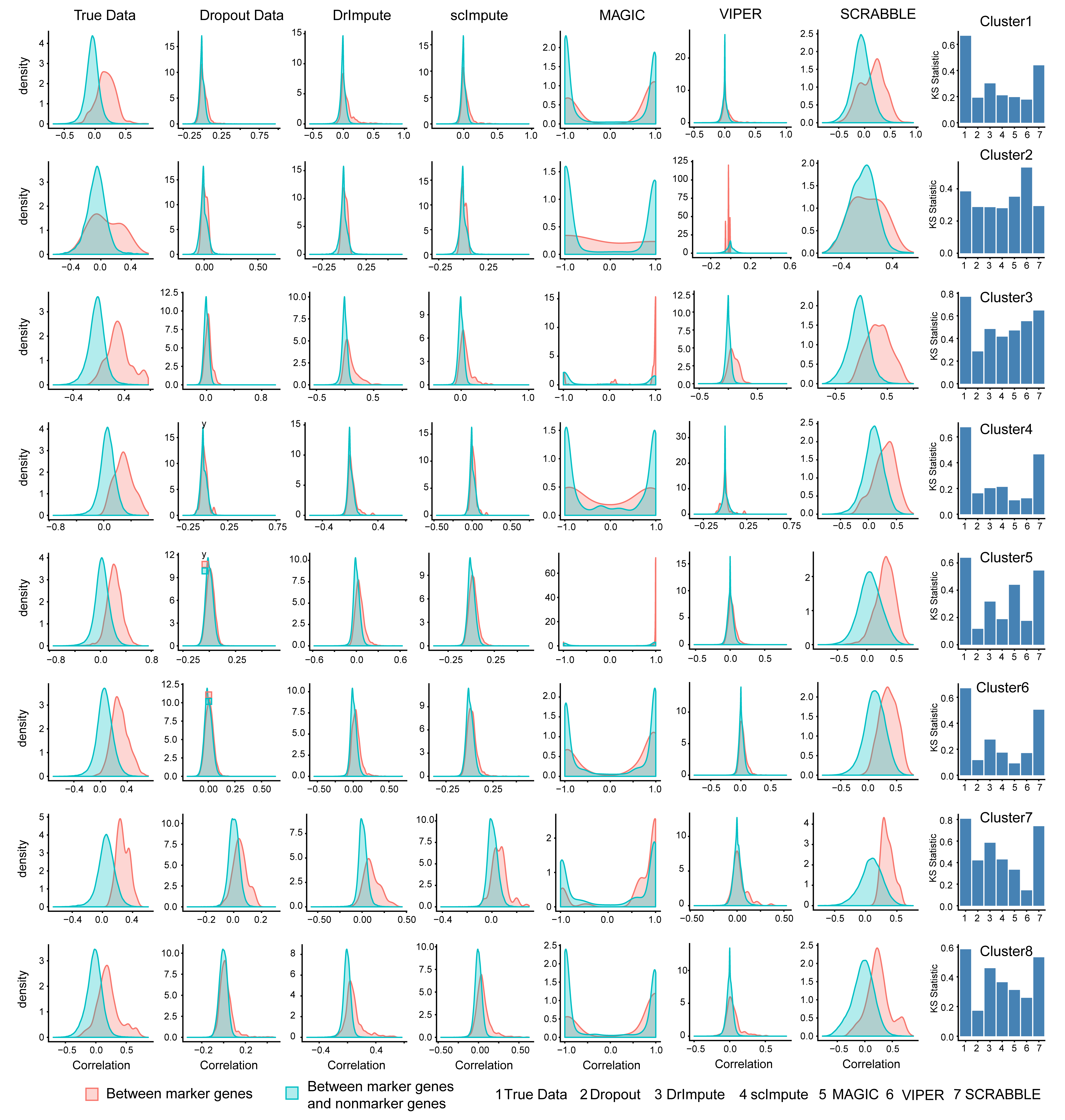


**Fig S20. Distance between marker and non-marker gene-gene correlations using data generated with simulation strategy 2 (related to Figure 5C and 5D)**. Distance between marker-marker and marker-non-marker correlation distributions is quantified using KS statistic. Values in each boxplot are the differences in the KS statistic between imputed and true data. Each boxplot is based on 100 sets of simulated data. P-values are based on Student’s t-test.


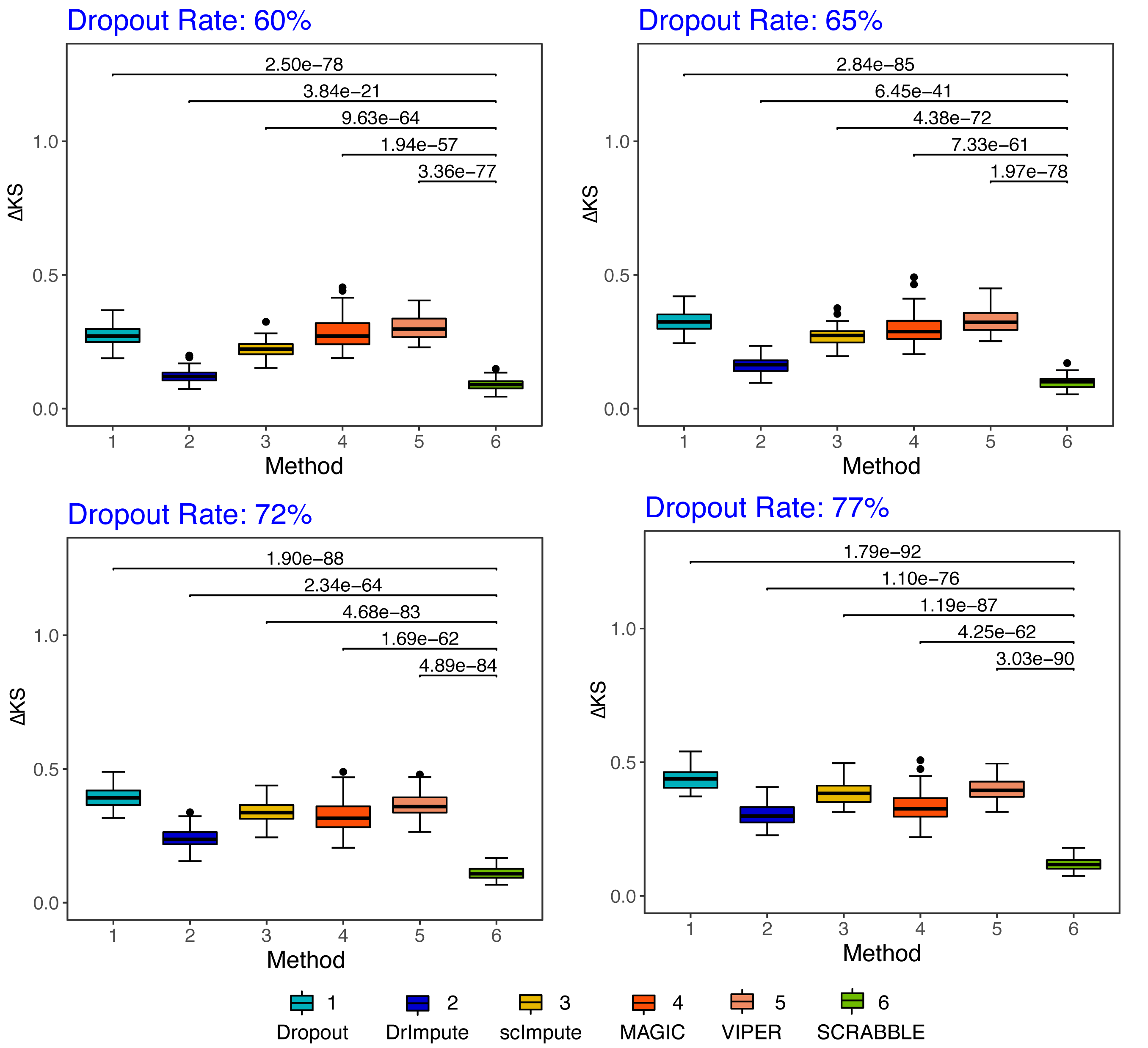


**Fig S21. Distance between within and between cluster cell-cell correlations using data generated with simulation strategy 1 (related to Figure 5A and 5B)**. Distance between within and between cluster correlation is quantified using KS statistic. Values in each boxplot are the differences in the KS statistic between imputed and true data. Each boxplot is based on 100 sets of simulated data. P-values are based on Student’s t-test.

**Fig S22. Distance between marker and non-marker gene-gene correlations using data generated with simulation strategy 1 (related to Figure 5C and 5D)**. Distance between marker-marker and marker-non-marker correlation distributions is quantified using KS statistic. Values in each boxplot are the differences in the KS statistic between imputed and true data. Each boxplot is based on 100 sets of simulated data. P-values are based on Student’s t-test.

**
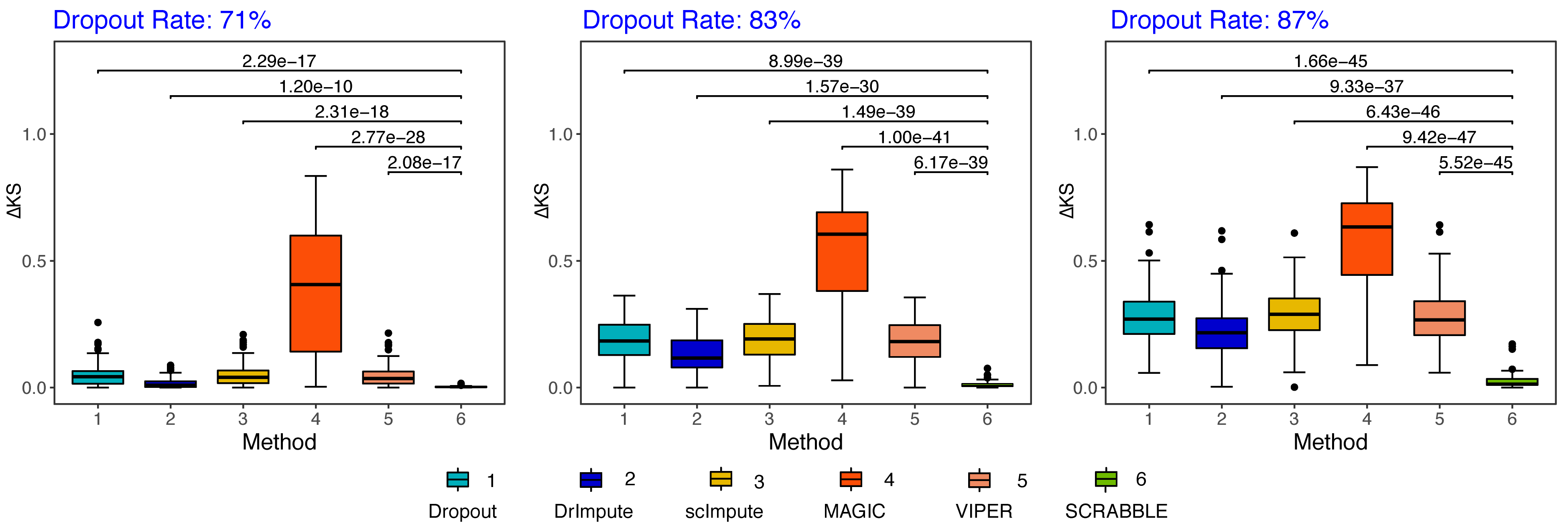
**

**Fig S23. Evaluation of gene-gene correlation based on imputed data using IPA pathway annotations (related to Figure 6).** Results for four additional cell types are shown, H9, human ES cells; DEC, definitive endoderm cells; EC, endothelial cells; NPC, neuronal progenitor cells. P-values are based on Student’s t-test.

**
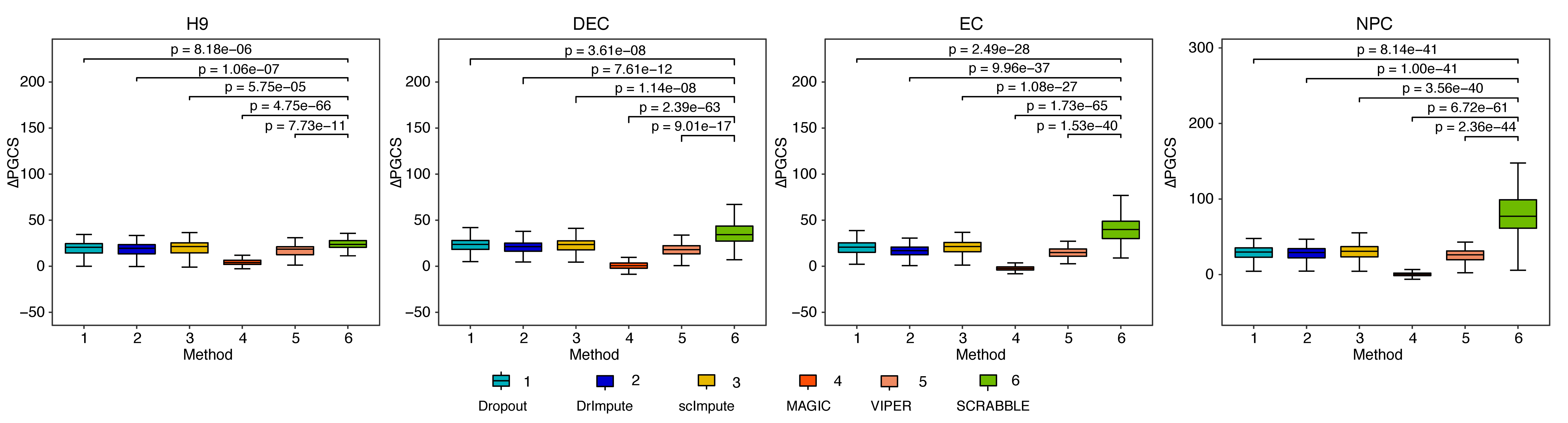
**

**Fig S24. Evaluation of gene-gene correlation based on imputed data using KEGG pathway annotations (related to Figure 6).** P-values are based on Student’s t-test.

**
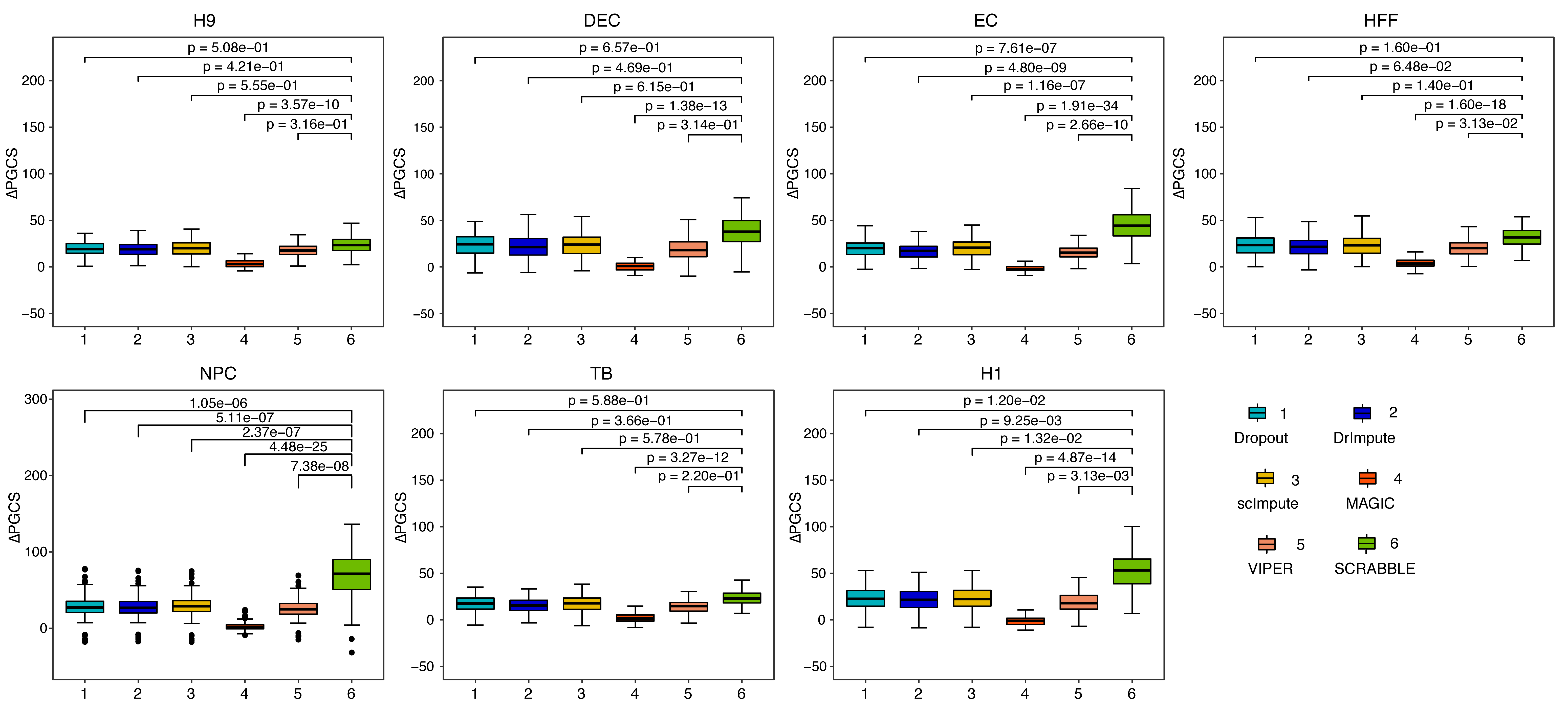
**

**Fig S25. Evaluation of gene-gene correlation based on imputed data using REACTOME pathway annotations (related to Figure 6).** P-values are based on Student’s t-test.

**
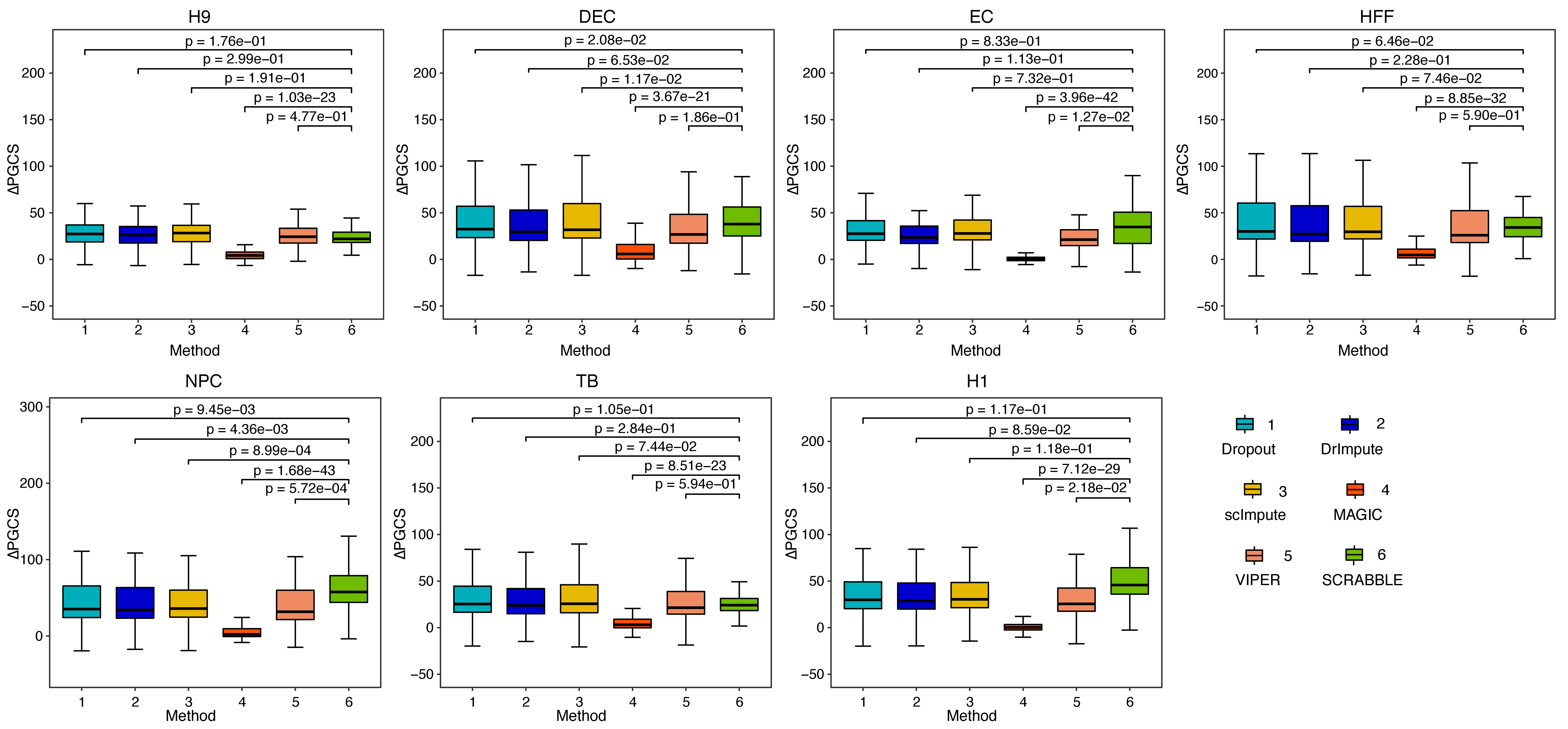
**

**Fig S26. Identification of cell types in mouse fetal liver using imputed data (related to Figure 7).** (**A**) Clustering results using un-imputed and imputed data by various methods. scRNA-Seq data was clustered using K nearest neighbor clustering and visualized using t-SNE. The number of clusters (K) was based on the ones provided by the authors. The cell type associated with each cluster was identified based on marker genes provided by the authors. (**B**) Quantification of cluster quality using the Dunn index.

**
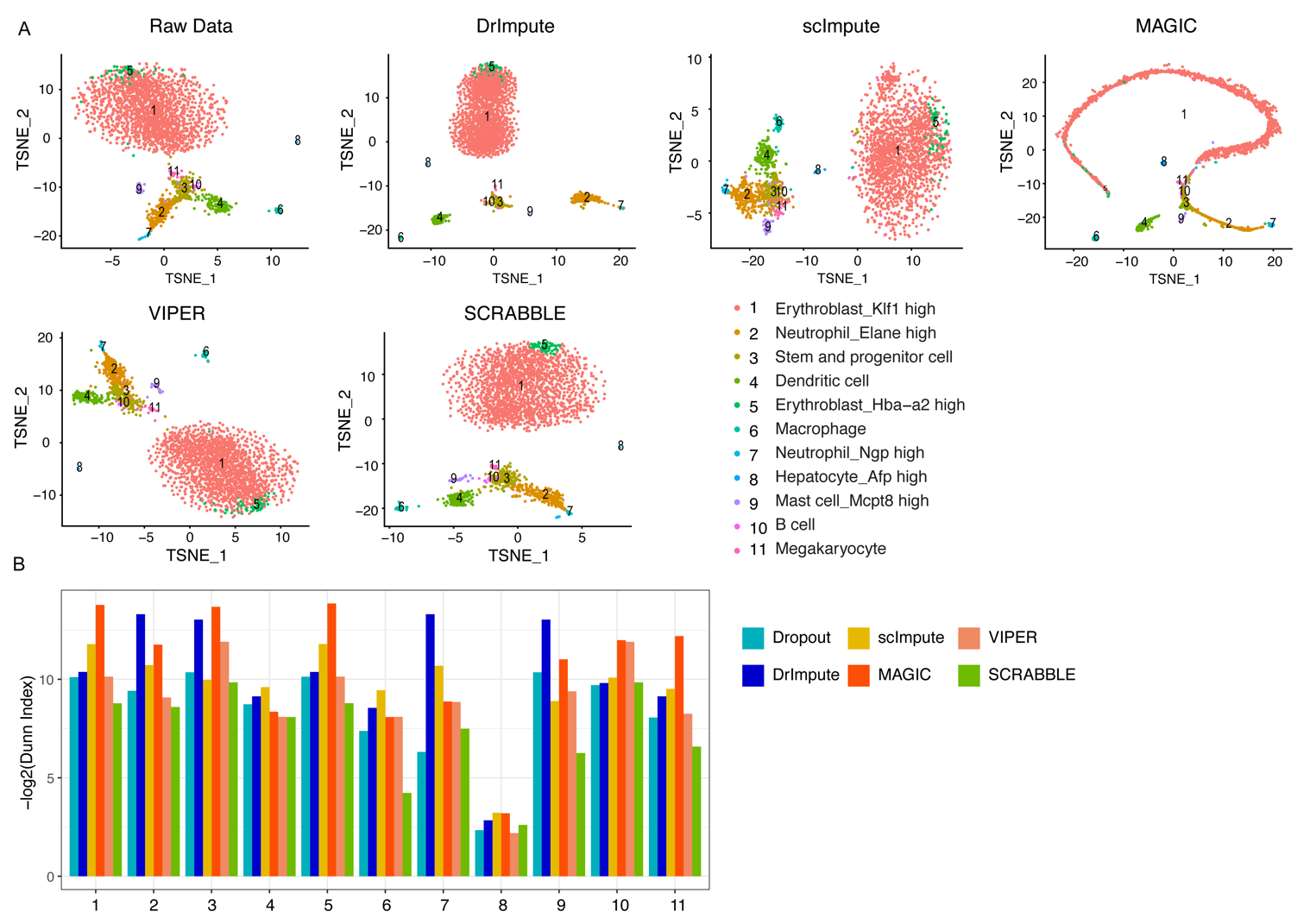
**

**Fig S27. Identification of cell types in mouse kidney using imputed data (related to Figure 7).**

**
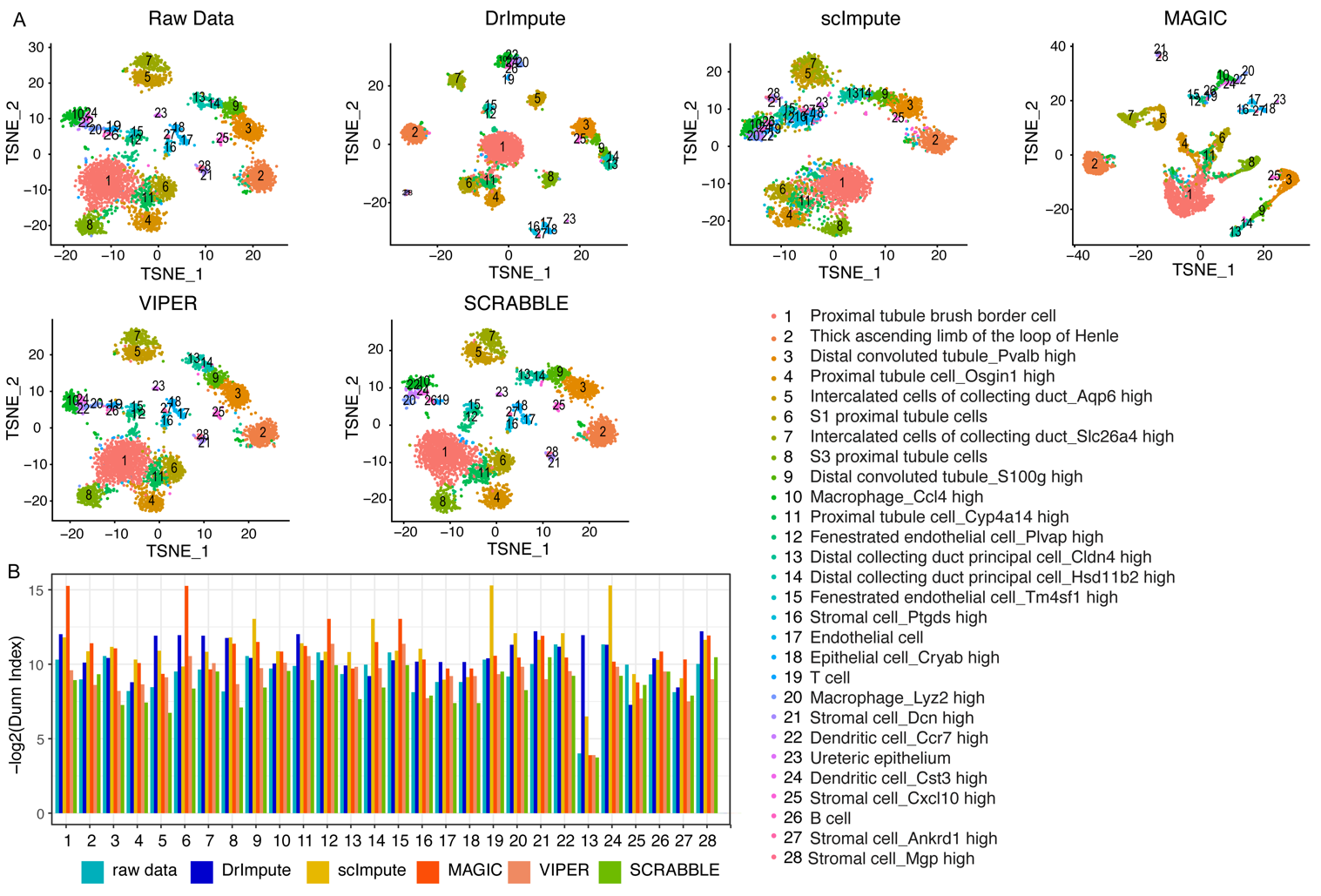
**

**Fig S28. Identification of cell types in mouse liver using imputed data (related to Figure 7).**

**
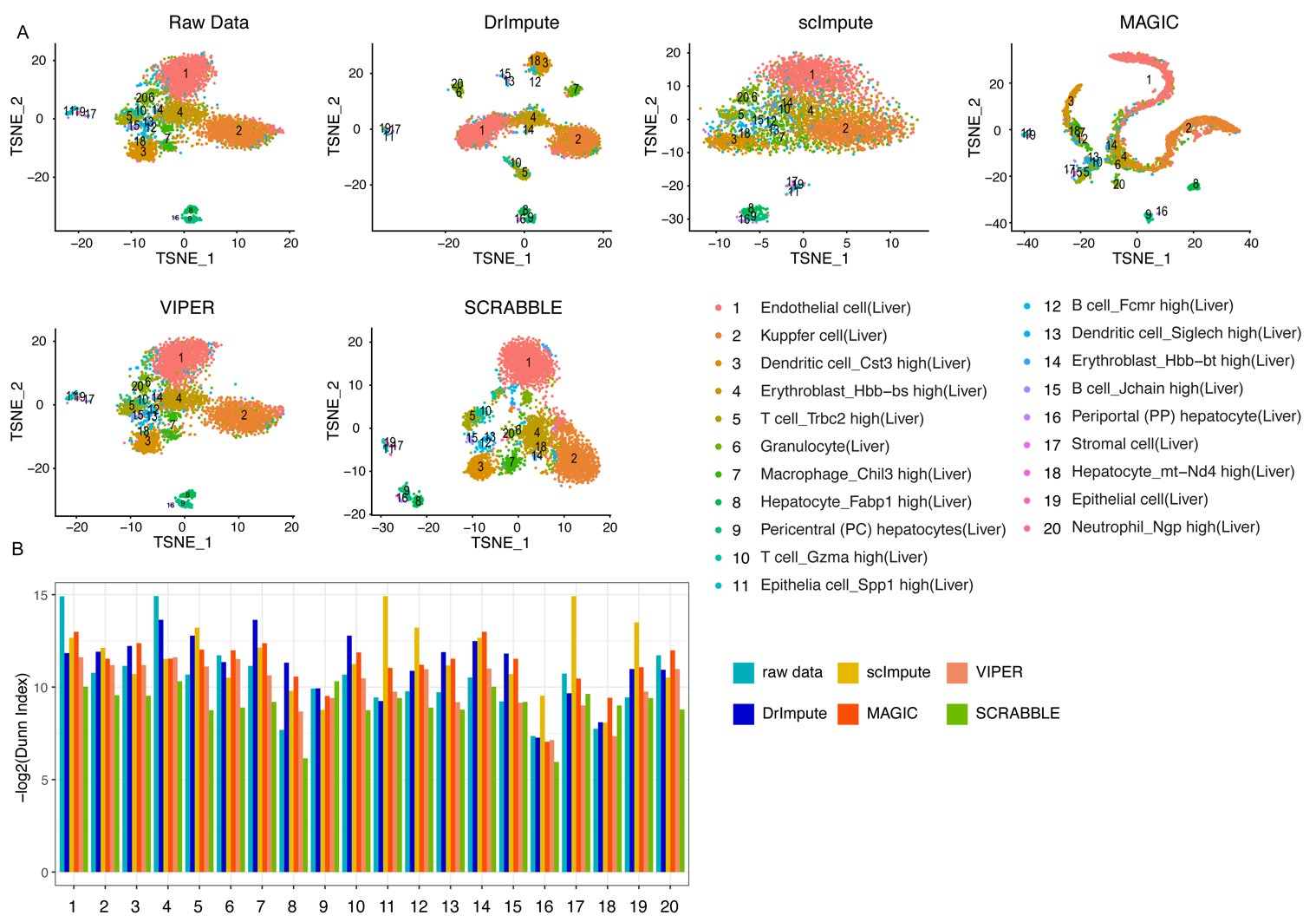
**

**Fig S29. Identification of cell types in mouse lung using imputed data (related to Figure 7).**

**
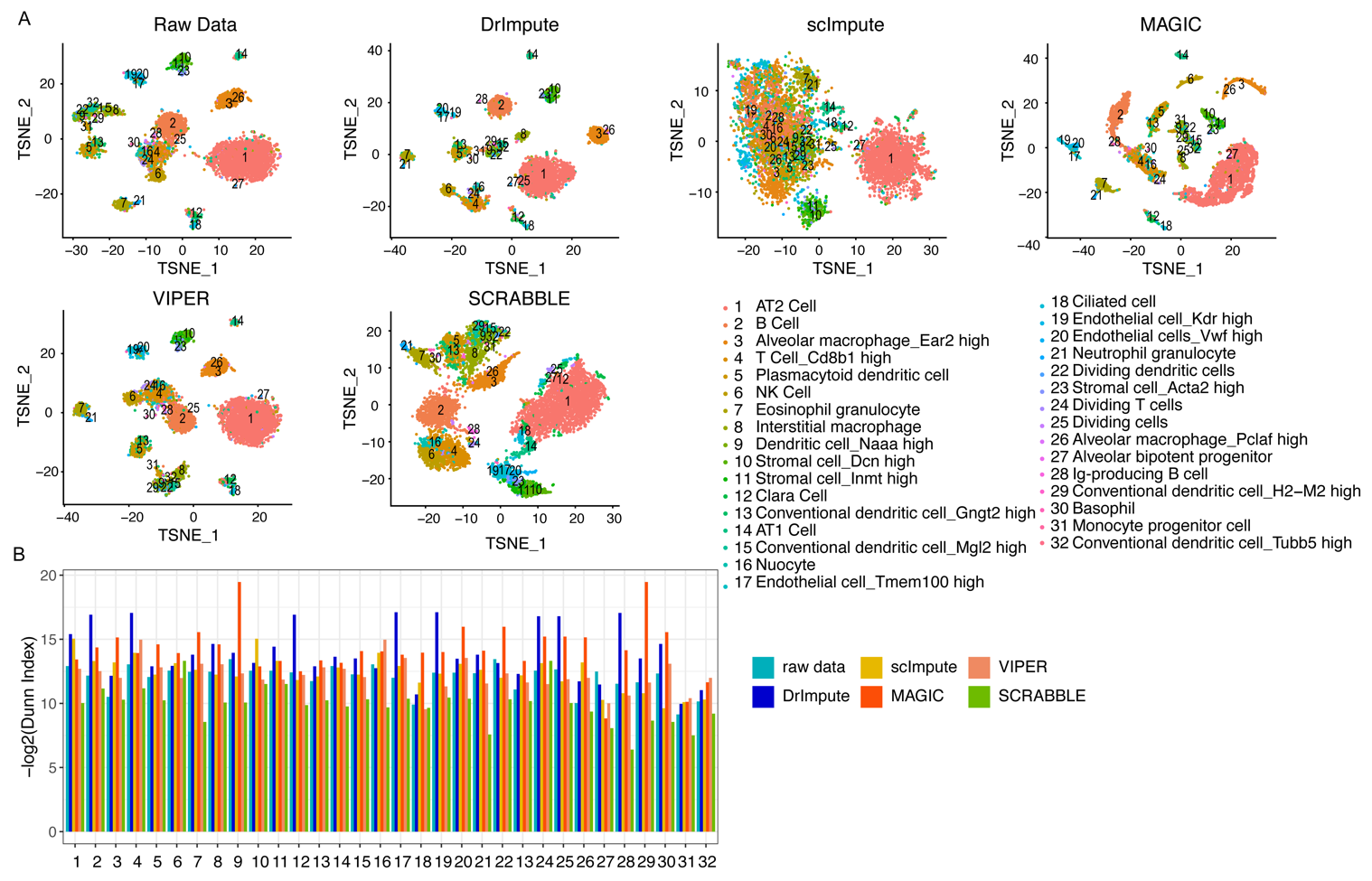
**

**Fig 30. Identification of cell types in mouse placenta using imputed data (related to Figure 7).**

**
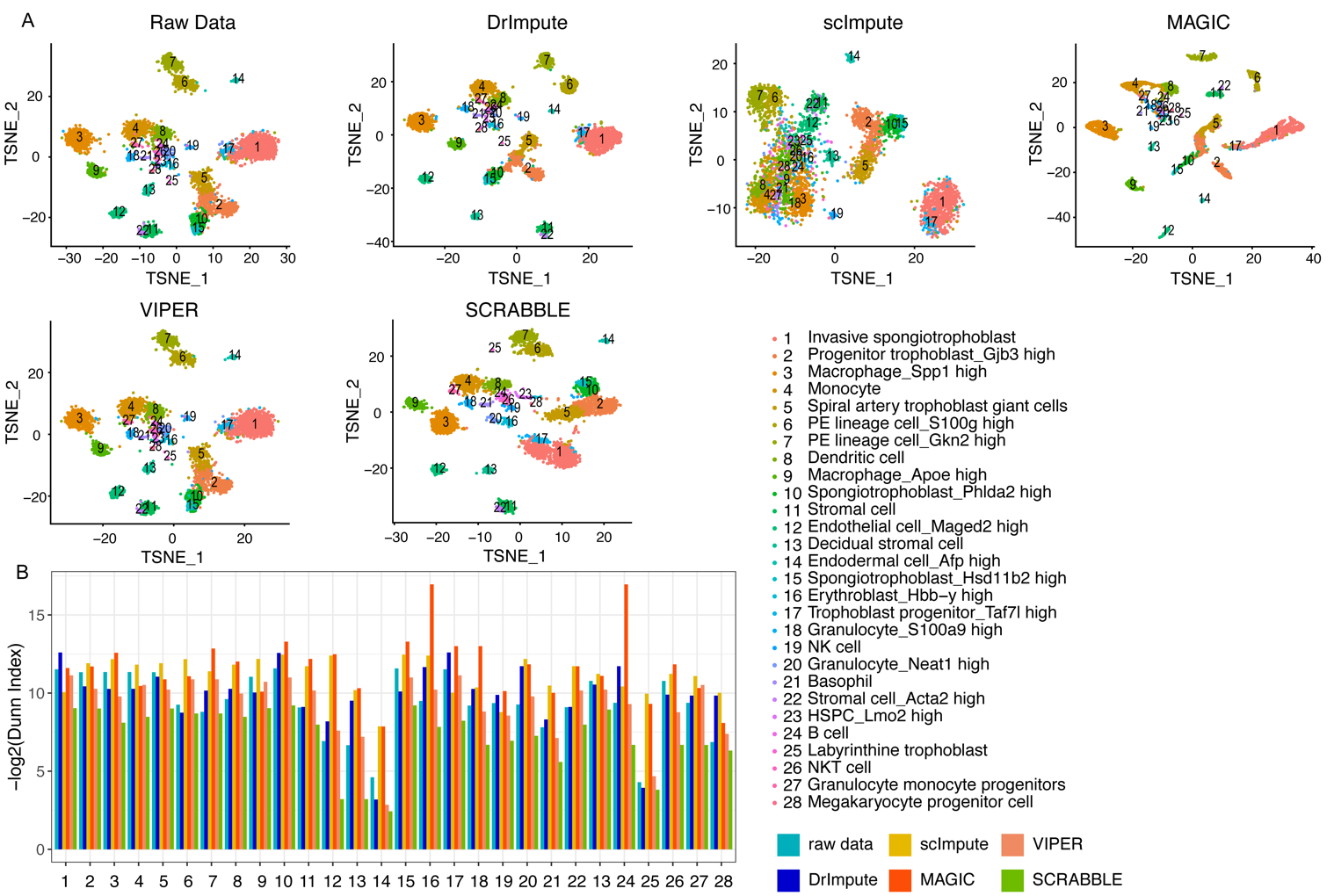
**

**Fig S31. Identification of cell types in mouse small intestine using imputed data (related to Figure 7).**

**
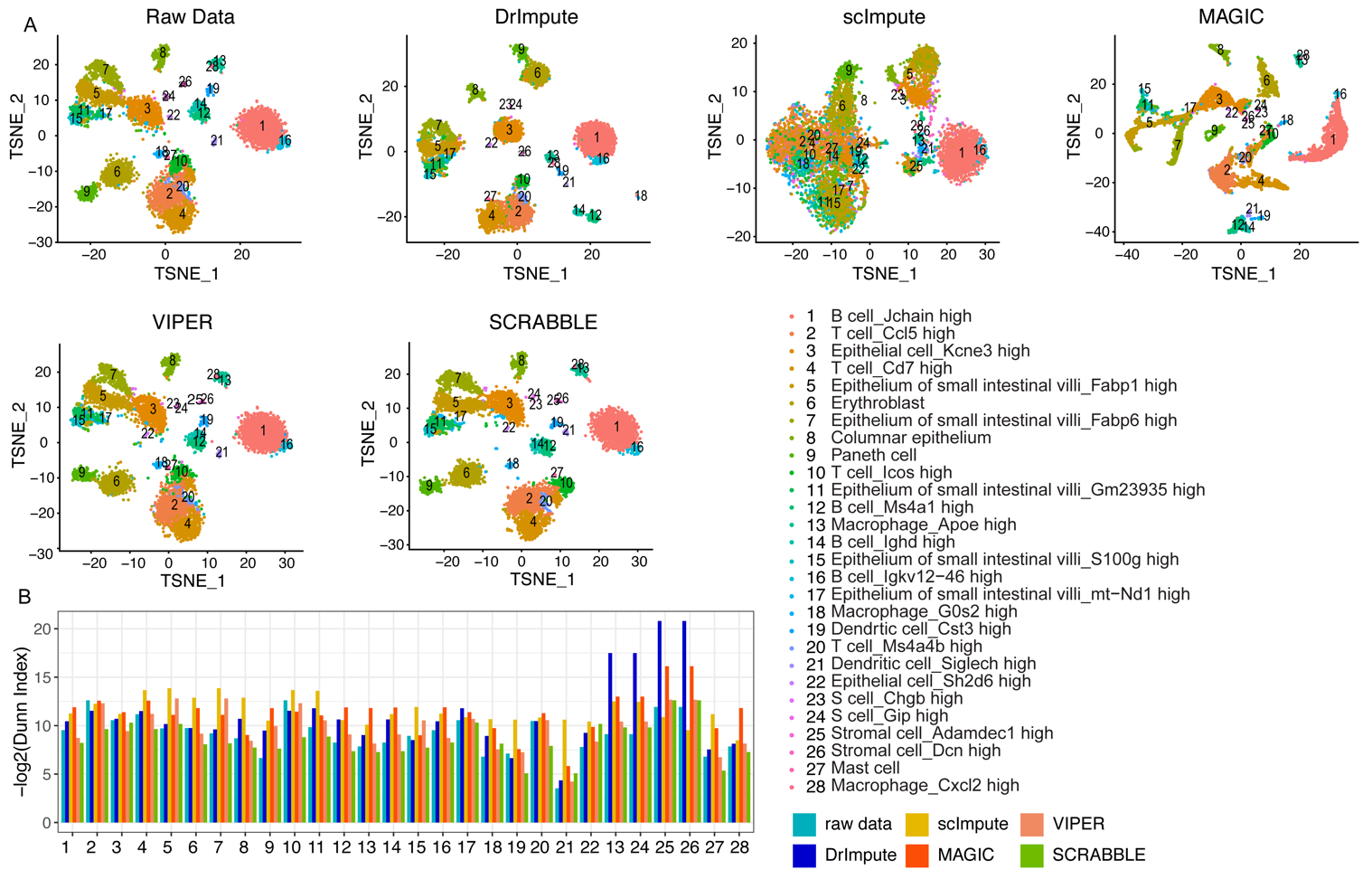
**

**Fig S32. Identification of cell types in mouse spleen using imputed data (related to Figure 7).**

**
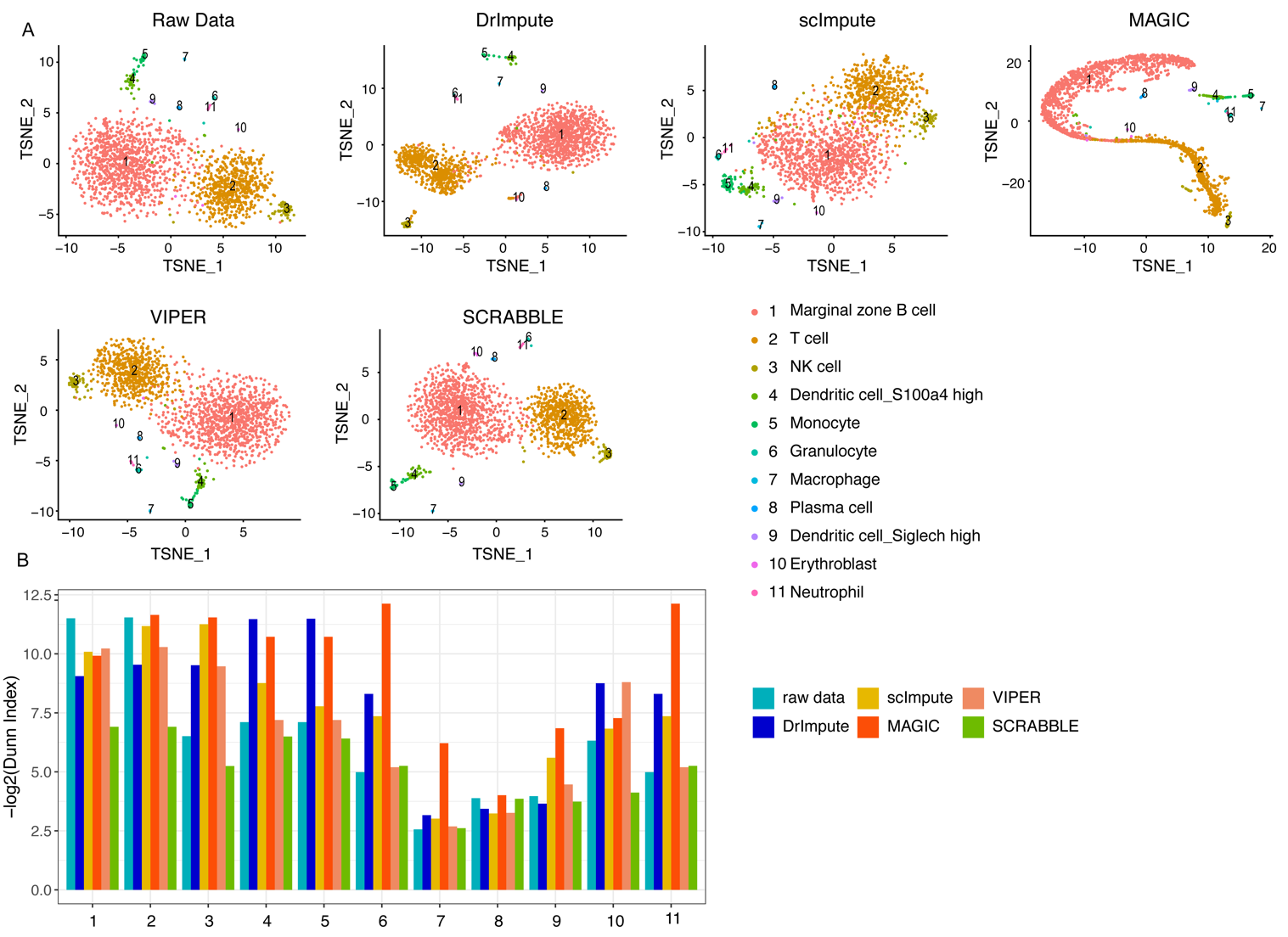
**

**Fig S33. Robustness analysis of SCRABBLE over parameter settings.** Data generated using simulation strategy 1 and drop-out rate of 83% was used**.** Y axis is the percent change in imputed data before and after changing the parameter. Each boxplot represents 100 simulated datasets. (**A**) Percent change at different values of the α parameter. Value of the parameter value was varied by 0.1, 0.5, 2, and 10 folds. (**B**) Percent change at different values of the β parameter. Value of the parameter value was varied by 0.1, 0.5, 2, and 10 folds. (**C**) Percent change at different values of the γ parameter. Value of the parameter was varied by 0.1, 0.5, 2, and 10 folds.

**Figure S34. Running time analysis.** All methods were run on a Dell workstation with an Intel Xeon CPU E5-2640 v4 with a clock speed of 2.40GHz. Each dataset had 1000 genes and varying number of cells. The running time for each method represents the mean value of 10 independent runs.


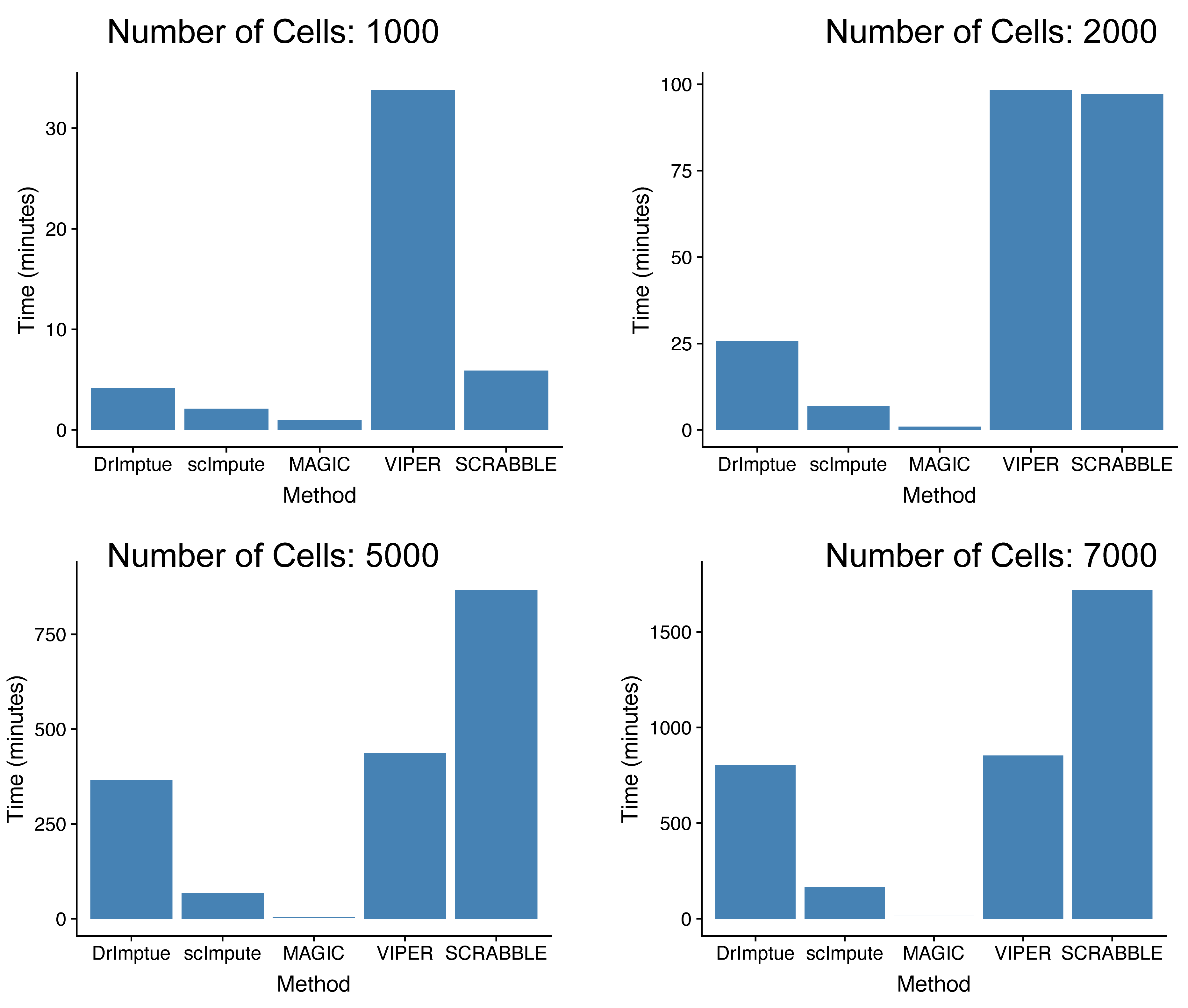

Supplement: Supplementary file 2 — Figures S7-S34. Supplementary figures. (DOCX 9400 kb) [file 13059_2019_1681_MOESM2_ESM.docx]
