# Supplementary material for: SCRABBLE: single-cell RNA-seq imputation constrained by bulk RNA-seq data
Source: Genome Biol. 2019 May 6;20:88. doi: 10.1186/s13059-019-1681-8 (PMC6501316; doi:10.1186/s13059-019-1681-8)
Supplement: Supplementary file 3 — Tables S1 and S2. Supplementary tables. (PDF 41 kb) [file 13059_2019_1681_MOESM3_ESM.pdf]

Supplementary Tables

Table S1. RNA-Seq data sources and literature references.

**Cell/Tissue Type**	**Data Type**	**GEO Accession Number**	**Pubmed ID**
Mouse hair follicle	Bulk RNA-Seq	GSE85039	PMID: 28695824
Mouse hair follicle	Single cell RNA-Seq	GSE85039	PMID: 28695824
Human ES cells	Bulk RNA-Seq	GSE75748	PMID:27534536
Human ES cells	Single cell RNA-Seq	GSE75748	PMID:27534536
Human lineage-specific progenitor cells	Bulk RNA-Seq	GSE75748	PMID:27534536
Human lineage-specific progenitor cells	Single cell RNA-Seq	GSE75748	PMID:27534536
Mouse ES cells (J1 line)	Bulk RNA-Seq	GSM2177789	PMID:21628449
Mouse ES cells (J1 line)	Single cell RNA-Seq	GSE75790	PMID:28212749
Mouse ES cells (E14 line)	Bulk RNA-Seq	GSE79578	PMID:29061959
Mouse ES cells (E14 line)	Single cell RNA-Seq	GSE79578	PMID:29061959

Table S2. Parameters used by SCRABBLE in the manuscript.

**Name of the analysis**	α	β	γ
Simulated data using Splatter (Strategy 1)	1	1.0e-6	1.0e-4
Simulated data using hair follicle data (Strategy 2)	1	1.0e-5	1.0e-2
Mouse ES cells (J1 line)	1	1.0e-7	1.0e-5
Mouse ES cells (E14 line)	1	1.0e-7	1.0e-5
Human lineage-specific progenitor cells	1	1.0e-5	1.0e-4
Mouse tissues: Spleen	1	1.0e-7	1
Mouse tissues: Fetal Brain	1	1.0e-7	1.0e-1
Mouse tissues: Small Intestine	1	1.0e-7	1
Mouse tissues: Kidney	1	1.0e-7	1
Mouse tissues: Liver	1	1.0e-7	1.0e-1
Mouse tissues: Fetal Liver	1	1.0e-7	1.0e-1
Mouse tissues: Lung	1	1.0e-7	1.0e-2
Mouse tissues: Placenta	1	1.0e-7	1.0e-1
